# Genetic Dissection of Sexual Reproduction in a Primary Homothallic Basidiomycete

**DOI:** 10.1371/journal.pgen.1006110

**Published:** 2016-06-21

**Authors:** Márcia David-Palma, José Paulo Sampaio, Paula Gonçalves

**Affiliations:** UCIBIO-REQUIMTE, Departamento de Ciências da Vida, Faculdade de Ciências e Tecnologia, Universidade Nova de Lisboa, Caparica, Portugal; Duke University Medical Center, UNITED STATES

## Abstract

In fungi belonging to the phylum Basidiomycota, sexual compatibility is usually determined by two genetically unlinked *MAT* loci, one of which encodes one or more pheromone receptors (P/R) and pheromone precursors, and the other comprehends at least one pair of divergently transcribed genes encoding homeodomain (HD) transcription factors. Most species are heterothallic, meaning that sexual reproduction requires mating between two sexually compatible individuals harboring different alleles at both *MAT* loci. However, some species are known to be homothallic, one individual being capable of completing the sexual cycle without mating with a genetically distinct partner. While the molecular underpinnings of the heterothallic life cycles of several basidiomycete model species have been dissected in great detail, much less is known concerning the molecular basis for homothallism. Following the discovery in available draft genomes of the homothallic basidiomycetous yeast *Phaffia rhodozyma* of P/R and HD genes, we employed available genetic tools to determine their role in sexual development. Two P/R clusters, each harboring one pheromone receptor and one pheromone precursor gene were found in close vicinity of each other and were shown to form two redundant P/R pairs, each receptor being activated by the pheromone encoded by the most distal pheromone precursor gene. The *HD* locus is apparently genetically unlinked to the *P/R* locus and encodes a single pair of divergently transcribed HD1 and HD2 transcription factors, both required for normal completion of the sexual cycle. Given the genetic makeup of *P*. *rhodozyma MAT* loci, we postulate that it is a primarily homothallic organism and we propose a model for the interplay of molecular interactions required for sexual development in this species. *Phaffia rhodozyma* is considered one of the most promising microbial source of the carotenoid astaxanthin. Further development of this yeast as an industrial organism will benefit from new insights regarding its sexual reproduction system.

## Author Summary

Some fungi are capable of sexual reproduction without the need for a sexually compatible partner, a behavior called homothallism. For some of these fungi, it was observed that they carried in a single individual all the genes normally determining sexual identity in two distinct sexually compatible individuals, but in most cases the role of these genes is still unclear. Here we examined in detail the homothallic sexual cycle of the yeast *Phaffia rhodozyma* that belongs to the Basidiomycota, which is the fungal lineage that also includes the mushrooms. *Phaffia rhodozyma* produces astaxanthin, a pigment with antioxidant properties used in the food and cosmetic industries and is accessible to genetic modifications, so far aimed mainly at improving astaxanthin production. Here we harnessed these genetic tools to dissect the self-fertile life cycle of this yeast and found that all genes normally involved in two-partner sexual reproduction are also required for self-fertile sex in *P*. *rhodozyma* and propose a model describing molecular interactions required to trigger sexual development. We also generated preferably outcrossing strains, which are potentially useful for further improvement of *P*. *rhodozyma* as an industrial organism.

## Introduction

The ability to reproduce sexually is a widespread trait in all eukaryotic lineages and it is pervasive in fungi, where key regulators of sexual behavior are encoded in specialized chromosomal regions named *MAT* loci [1]. Mating in fungi is governed by mechanisms of self/non-self recognition, and it usually involves two individuals that carry different alleles at the respective *MAT* loci and thus belong to distinct mating types. A feature common so far to all fungal mating systems seems to be the involvement of mating type specific transcription factors that contribute to post-mating sexual development [1]. In Ascomycota and in the early derived Zygomycota, *MAT* identity is defined at a single locus that encodes one of two possible, very dissimilar idiomorphic sequences. Hence, in these two lineages, mating systems have so far been found to be bipolar, which means that that they have a single *MAT* locus and spores belonging to two distinct mating types may arise after meiosis [1,2]. While much remains to be unraveled concerning the genetics of sex systems in the Zygomycota, in the Ascomycetes it is well known that two types of pheromone receptors (dubbed Ste2 and Ste3 in the model yeast *Saccharomyces cerevisiae* [3,4]), and cognate pheromones, are encoded in the genomes of both mating types outside the *MAT* locus. Each type of haploid cell expresses only one type of pheromone and one type of pheromone receptor, which define their sexual identity and promote mate recognition and cell fusion [5–7].

The Basidiomycota form together with the Ascomycota a lineage of derived fungi named Dikarya. Interestingly, early in the evolution the Basidiomycota, which include many fungi relevant for human activity like the mushrooms and the human pathogen *Cryptococcus neoformans*, important modifications occurred in the mechanisms of mating type determination that originated the so called tetrapolar mating system [8–10]. In the tetrapolar system, mating type is determined by two genetically unlinked *MAT* loci, so that after meiosis four different mating types resulting from different combinations of the two *MAT* loci may form. The tetrapolar system is currently thought to be the ancestral mating system in this phylum [8,9,11]. One distinctive feature of the tetrapolar system resides in the mating type defining role acquired by genes encoding pheromones and pheromone receptors. In Basidiomycota the Ste2 type of receptor was apparently lost and different Ste3 type alleles contribute to define mating type identity and are usually encoded in one of the two *MAT* loci [8,12]. Hence, contrarily to what happens for example in *S*. *cerevisiae*, the contribution of receptors to define the mating type is determined by which pheromone receptor and pheromone precursor alleles are encoded in one of the two *MAT* loci, rather than by their differential expression in opposite mating types. In many basidiomycetes, the pheromone/receptor compatibility between two potential mating partners is a pre-requisite for cell fusion [1,9,13].

The second important distinctive feature of basidiomycete mating systems is the involvement of a pair of divergently transcribed genes encoding homeodomain transcription factors, named *HD1* and *HD2* encoded in the second *MAT* locus, which constitutes another checkpoint for self/non-self recognition in addition to the pheromone/receptor (P/R) system [1,8,9]. In the species where this has been studied in detail, the HD1 and HD2 proteins potentially form a heterodimer which is a key regulator of post mating sexual development that usually involves morphological changes like the formation of dikaryotic mycelium, and culminates in the development of basidia and basidiospores [1,9,14]. However, dimerization only occurs between HD1 and HD2 proteins originating from individuals of different mating types [14,15]. Hence, haploid cells do not produce functional HD1/HD2 heterodimers, which can only form after fusion of compatible mating partners. The P/R and HD1/HD2 recognition systems function independently so that successful mating only occurs when both compatibility hurdles are overcome [1,8,9]. The tetrapolar system favors outcrossing because it decreases the chances of mating compatibility between sibling spores (25%), while the prevalent bipolar mating type system in the Ascomycetes facilitates inbreeding with a 50% compatibility chance [9,16]. Moreover, the tetrapolar system is often associated with multiallelism at both loci resulting in the generation of up to thousands of mating types as observed for some mushrooms [9,16,17].

The architecture of fungal sexual reproduction systems is obviously directed primarily at governing mating between genetically distinct individuals, usually referred to as heterothallism. However, very early on, mycologists noted that some fungi are capable of completing the sexual cycle without the need for a genetically distinct partner [18,19]. Homothallism is seen as possibly favorable in that it ensures important advantages of sex, like purging of deleterious mutations, while circumventing the necessity to find a compatible mating partner. It should be emphasized that homothallism admits outcrossing as well as inbreeding, and in one instance it was even proposed to favor outcrossing [20]. Homothallism is found across all fungal lineages and its morphological manifestations are in all cases the appearance of sexual structures in cultures derived from a single individual [1,9,18,19]. However, in spite of the apparent similitude of different instances of homothallism, the advent of molecular genetics, and more recently of comparative genomics, unraveled profound differences between the underlying molecular mechanisms. Conceptually, the simplest form is primary homothallism, consisting in the co-occurrence in the genome of a single individual of all the *MAT* alleles required to trigger sexual development [19]. In basidiomycetes, this would require in principle the presence in a single individual of at least one compatible pheromone/pheromone receptor pair and at least one HD1/HD2 pair capable of forming a functional heterodimer, while in Ascomycetes it would entail the presence in a single genome of all the transcription factor genes present at the *MAT* loci of the two opposite mating types. Following the occurrence of an endoreplication event [21] or fusion with another cell, the resulting cells are in principle genetically fully equipped to undergo meiosis and complete the sexual cycle alone. Although several ascomycete species have been described in which the presumed genetic prerequisites of primary homothallism are present (e. g. *Aspergillus nidulans*) [22,23], the role of *MAT* genes in the respective homothallic life cycle has not been fully elucidated so far [24–26]. Primary homothallism has been proposed for a handful of species in the Basidiomycota, most notably *Sistotrema brinkmannii* [27] and the C- biotype of the cacao pathogen *Moniliophtora perniciosa* [28]. Other forms of homothallism, often mechanistically very complex, have been uncovered over the years, the best studied of which is the pseudo-homothallic life cycle of *S*. *cerevisiae* that is capable of mating type switching [1,2,5]. More recently another particular form of homothallism dubbed unisexual mating (a.k.a. homokaryotic fruiting) was described in several fungi belonging to both lineages of Dikarya, most notably the human pathogens *C*. *neoformans* [29,30] and *Candida albicans* [30]. However, these mechanisms are clearly distinct from primary homothallism in many aspects, including the fact that species where pseudo-homothallic or unisexual mating have been identified harbor different mating types [29–31].

In the present work we undertook the genetic dissection of the homothallic life cycle of *Phaffia rhodozyma*, an orange pigmented astaxanthin producing yeast belonging to the Agaricomycotina, a major clade of Basidiomycota that also includes *C*. *neoformans* and the mushrooms. Astaxanthin is a carotenoid of significant economic value, used in the food and cosmetic industries because of its antioxidant activity and in aquaculture feed as a source of pigment [32]. *Phaffia rhodozyma* is readily transformable and its biotechnological potential fostered the development of molecular genetic tools that have been extensively used to improve astaxanthin production [33,34]. We recently uncovered a diversity hotspot of *P*. *rhodozyma* in the Southern hemisphere [35] and identified four populations within the species, which seem to correlate with different host trees [35] and two new, more distantly related lineages proposed to represent new *Phaffia* species [35]. The new species resemble *P*. *rhodozyma* in that all individuals are homothallic [35,36]. This means that all individual strains within each of the three *Phaffia* species presently considered are capable of originating typically slender basidia that usually develop four basidiospores at the apex when cultivated on medium containing polyols as sole carbon source [37]. Three types of structures (S1 Fig) were observed to originate the formation of basidia in *P*. *rhodozyma*, the most common being conjugation between a cell and its bud (a.k.a. pedogamy) [37]. However, basidia were also observed to originate from unconjugated cells where possibly endoreplication precedes meiosis as observed in other fungi [21] and from independent cells that conjugate prior to sexual development [36–38]. Similar observations were previously reported for strains belonging to the two proposed new *Phaffia* species [35]. Hence, as far as can be presently ascertained, the lineage corresponding to the entire genus *Phaffia* consists entirely of homothallic species, wherein no heterothallic individuals have been identified [35].

Recently, inspection of the genomes of three *P*. *rhodozyma* strains [39] (Nicolás Bellora and Diego Libkind, personal communication) revealed the presence of pheromone precursor and pheromone receptor (P/R) gene clusters and one HD1/HD2 pair similar to those commonly found in heterothallic basidiomycete *MAT* loci. The two pheromone receptor genes exhibit considerable sequence divergence and each is flanked by a unique pheromone precursor gene. The *P/R* locus is seemingly genetically unlinked to the *HD* locus, since in all *P*. *rhodozyma* draft genome assemblies examined the P/R and HD genes are located on different scaffolds. The identification of the above mentioned *MAT* related genes raised the question of whether they have a role in the homothallic life cycle of *P*. *rhodozyma*. In the present study we first examined, and were able to discard, the possibility that cryptic molecular mating types might exist among available *P*. *rhodozyma* strains, in spite of their homothallic behavior. We subsequently made use of genetic tools available for this species to undertake the dissection of the genetic underpinnings of sexual reproduction in *P*. *rhodozyma*. Although ploidy of *P*. *rhodozyma* strains is thought to be diploid or higher in most cases [40], all genetic studies performed so far in strain CBS 6838 indicate that it is haploid [41]. This strain was, therefore, used as genetic background for the construction of various deletion mutants that contributed to show that all six *MAT* genes identified seem to be functional and to play a role in sexual reproduction. Taken together, these results allowed us to propose for the first time a mechanistic model for primary homothallism in a basidiomycete.

## Results

### No evidence for different molecular mating types in *P*. *rhodozyma*

**Fig 1:**
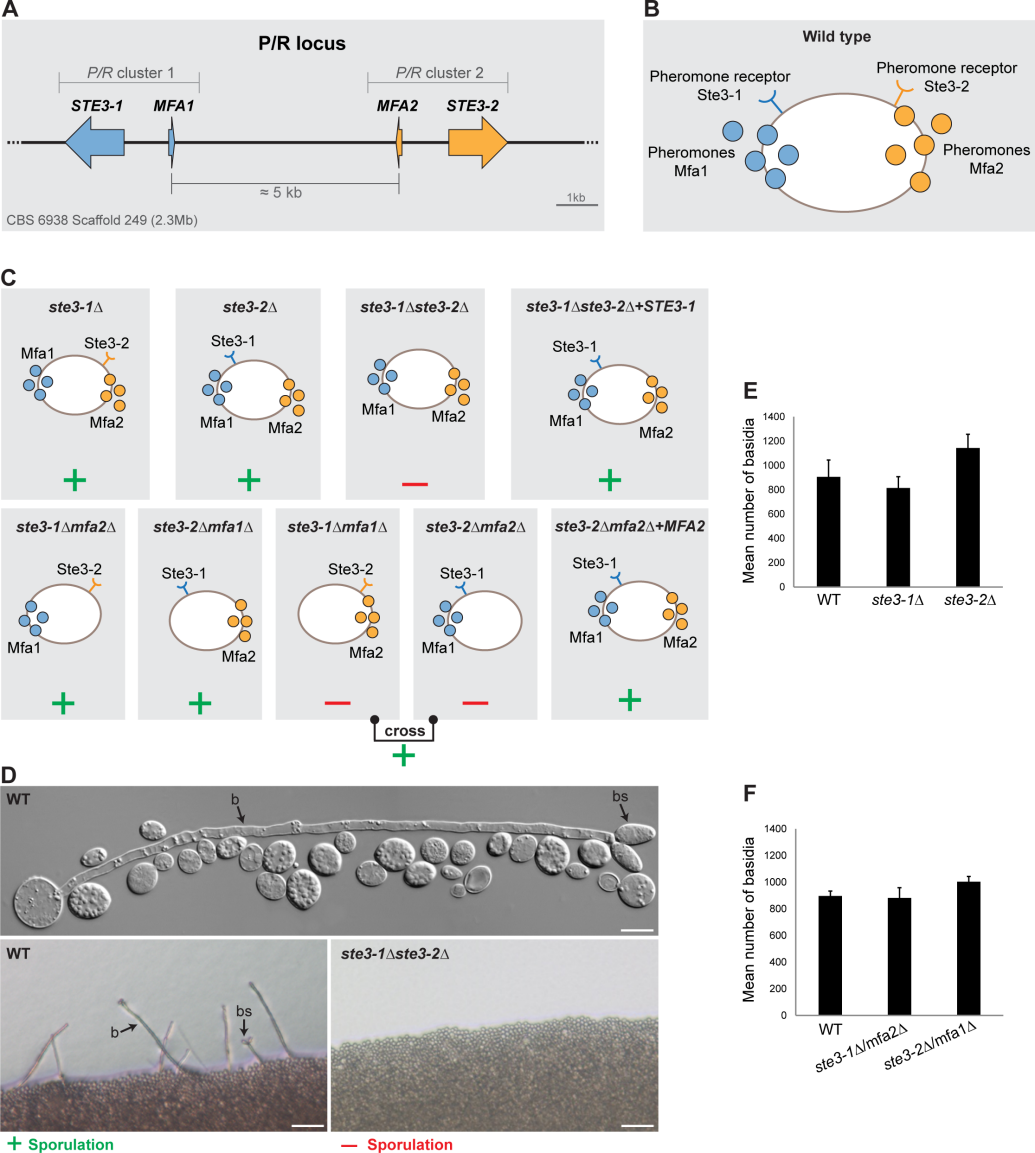
Deletion mutants in the P/R locus. **A.** Organization of the *P/R* locus of strain CBS 6938 depicting gene clusters P/R1 (blue) and P/R2 (orange). **B.** Pheromones and pheromone receptors as are expected to be expressed the wild type cells. **C.** *P/R* locus genes expressed and sporulation phenotypes of mutants in which one or more *P/R* locus genes have been deleted. Plus signs (dark green) indicate that formation of basidia and basidiospores was observed, while minus (red) signs denote complete failure of the mutant to sporulate. **D.** Light microscopy photographs showing an example of positive and negative sporulation phenotypes, as indicated. Letters b and bs denote basidium and basidiospore, respectively. **E.** and **F.** Mean number of basidia observed per plate in sporulation proficient mutants after 10 days of incubation in sporulation medium at 18°C. Error bars represent standard deviations from the mean for the three biological replicates. No significant differences were observed between the WT and the sporulation proficient mutants (Student’s T test).

The existence of a minimum of two *MAT* alleles or *MAT* idiomorphs among distinct individuals within a population, defining at least two compatible mating types, can be considered as indicative of the potential for heterothallism, which may co-exist with homothallism in some species [2]. In the tetrapolar (heterothallic) system, the *HD* genes are multiallelic (more than two alleles are found in the population of a given species) [1,9,16] while the pheromone precursor and pheromone receptor loci can be either multiallelic, as is the case in many mushroom species [9,12,42] or biallelic as in red yeasts [43] and the model smut fungus *Ustilago maydis* [8] among many others. In each of the three available genomes of *P*. *rhodozyma* [39] (Nicolás Bellora and Diego Libkind, personal communication) we identified two clusters encoding each a pheromone precursor and a Ste3 type receptor (henceforth referred to as *P/R1* and *P/R2*) located at approximately 5 kb from each other (Fig 1A). The Ste3 type receptors encoded in the P/R1 and P/R2 clusters are clearly different from each other (S2 Fig). The occurrence in the same genome of multiple pheromone receptor genes is quite common in heterothallic species within the Agaricomycotina. In several well-studied cases, different combinations of *P/R* clusters define distinct mating type identities [9,42]. To assess whether additional pheromone receptors alleles potentially encoding different mating identities could be retrieved in *P*. *rhodozyma*, we obtained partial sequences for the pheromone receptor genes found in the *P/R1* and *P/R2* clusters of 14 individuals representing all four previously identified populations of *P*. *rhodozyma* (S3 Fig) [35]. This survey uncovered 10 variants for both the *STE3-1* gene (from *P/R* cluster 1) and the *STE3-2* gene (from *P/R* cluster 2) that are nevertheless very similar within the cohort formed by genes originating from the same cluster (S3 Fig). A comparison of the three full length amino acid sequences of Ste3-1 and Ste3-2 available (S2 Fig) shows a maximum of seven and three amino acid substitutions between variants of Ste3-1 and Ste3-2, respectively. These differences are found between strains belonging to different populations (CRUB 1149 from population A vs. CBS 7918 and CBS 6838 from population C), while the two strains belonging to the same population (CBS 7918 and CBS 6838) exhibit identical amino acid sequences. We conclude that these variants are very unlikely to encode functionally different receptors. Both the *STE3-1* and *STE3-2* phylogenies including all the identified variants reproduce well the previously reported relationships between the phylogenetic lineages (populations) within *P*. *rhodozyma* (S3 Fig). For the genes encoding the pheromone precursors we did not conduct a broad population survey but a comparison of the genes present in the three genomes available that encompass two distinct *P*. *rhodozyma* populations (A and C; S2 Fig) also suggests a high similarity between sequences belonging to the same cluster. Hence, taken together, these results strongly indicate that the *P/R* locus is not bi- or multiallelic in *P*. *rhodozyma*, as opposed to heterothallic basidiomycetes.

**Fig 2:**
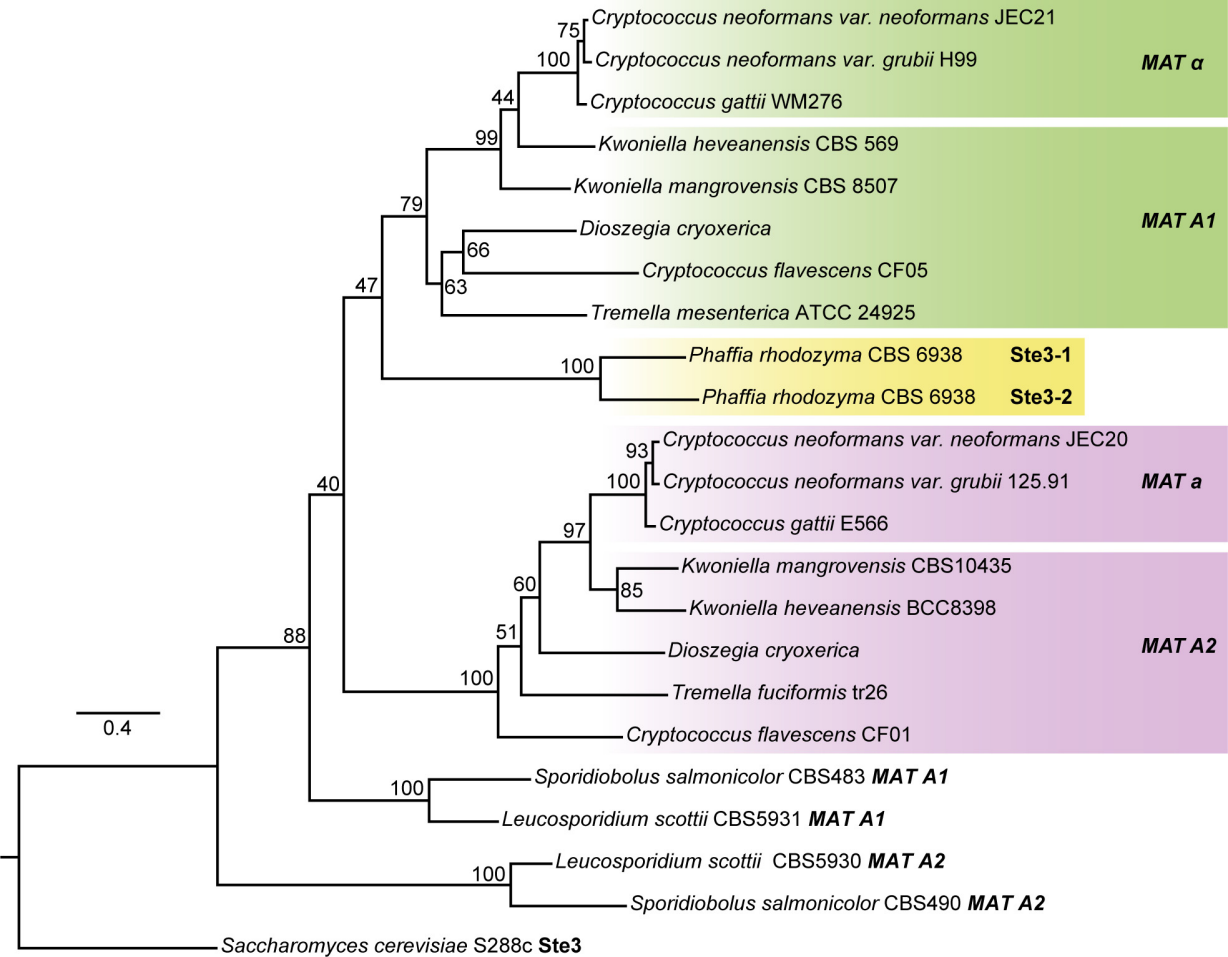
Maximum likelihood phylogenetic tree of pheromone receptors (Ste3) from Tremellomycetes. Phylogeny includes Ste3 protein sequences from the species within Tremellomycetes closest to *Phaffia* and is rooted with the sequences of both Pucciniomycotina species (*L*. *scottii* and *S*. *salmonicolor*) and *S*. *cerevisiae* Ste3 pheromone receptors.

Many heterothallic basidiomycete species, both bipolar like *Cryptococcus neoformans* [44] and *Ustilago hordei* [45] or tetrapolar, like *Leucosporidium scottii* [11] or *Ustilago maydis* [46] harbor two functionally distinct, and thus mating type determining, Ste3 receptors. When phylogenies are constructed using the amino acid sequences of these two pheromone receptors identified in various basidiomycete lineages, trans-specific polymorphism is usually observed, meaning that each of the two Ste3 receptors found in one species is phylogenetically more closely related to the receptor in the corresponding mating type of a different species than to its counterpart in the opposite mating type of the same species [9,47,48]. To find out how the two genes of *P*. *rhodozyma* fit in this general picture, a phylogenetic analysis was conducted including the Ste3-1 and Ste3-2 receptors of *P*. *rhodozyma* CBS 6938 and their closest known relatives from heterothallic species harboring two mating type specific Ste3 receptors, from the *Cryptococcus*, *Kwoniella* and *Tremella* lineages. While reproducing the expected trans-specific polymorphism for all other species examined, the phylogeny in Fig 2 shows that the two *P*. *rhodozyma* receptors share a more recent common ancestor with each other than with the receptors found in the other species and consequently do not exhibit trans-specific polymorphism. In line with this, the protein sequences of Ste3-1 and Ste3-2 receptors have diverged considerably less than the two Ste3 alleles found in the other (heterothallic) species examined (50% amino acid identity, S1 Table and S2 Fig). On the other hand, they seem to be more divergent than some alleles identified in heterothallic species like *Coprinopsis cinerea* possessing multiple functional receptor paralogs per genome that can be up to 80% identical while exhibiting different pheromone binding properties [42].

**Fig 3:**
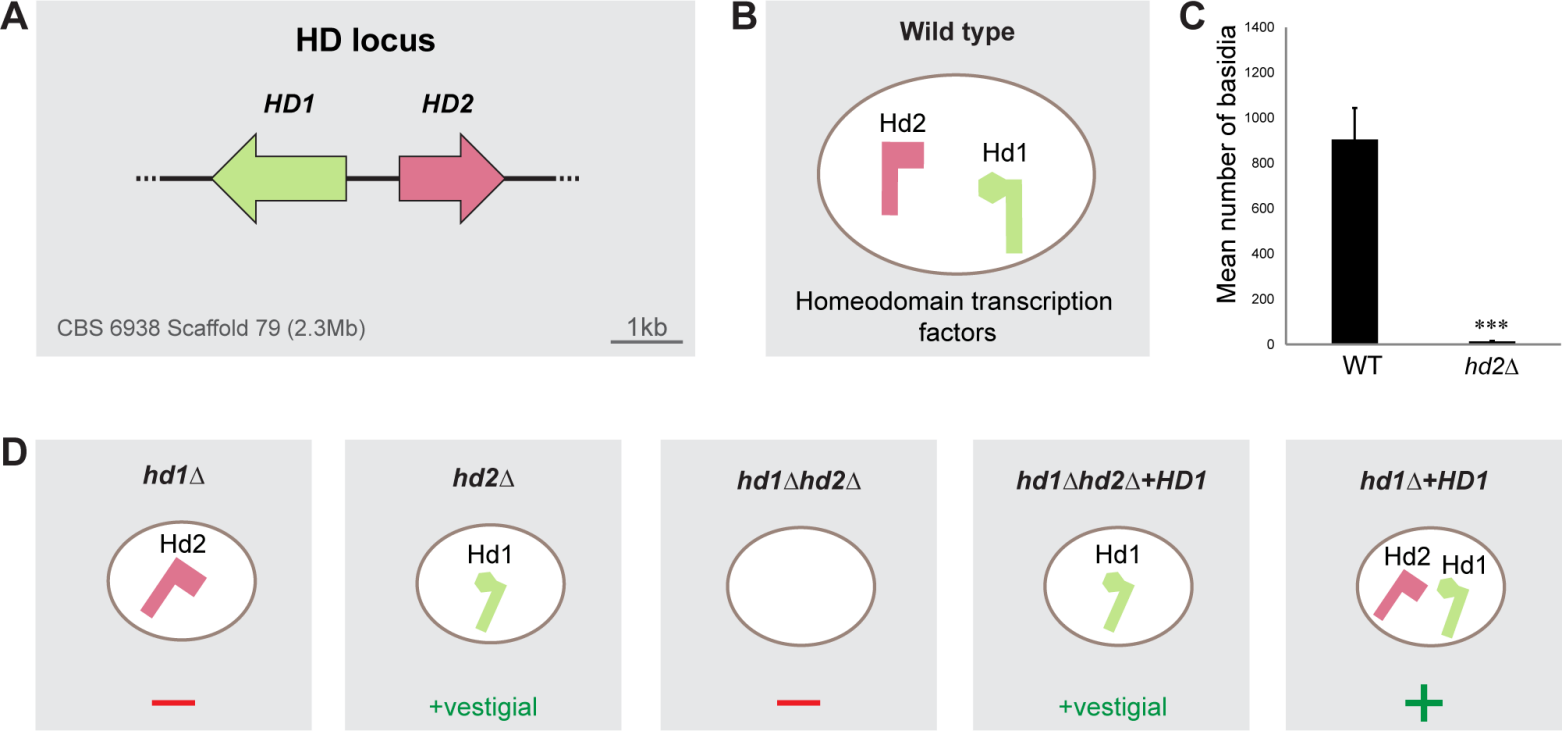
Deletion mutants in the HD locus. **A.** Organization of the *HD* locus of strain CBS 6938 depicting the HD1 (green) and HD2 (pink) genes. **B.** Homeodomain transcription factors as are expected to be expressed in wild type cells. **C.** *HD* locus genes expressed and sporulation phenotypes of mutants in which one or both *HD* locus genes have been deleted. Plus signs (dark green) indicate that formation of basidia and basidiospores was observed, while minus signs (red) denote complete failure of the mutant to sporulate. Smaller plus sign was used to denote that only vestigial sporulation was observed in mutants expressing only HD1. **D.** Number of basidia observed per plate in sporulation proficient mutants after 10 days of incubation in sporulation medium at 18°C. Error bars represent standard deviations from the mean for three biological replicates. Asterisks denote significant difference between the WT and the *hd2Δ* mutant (Student’s t-test, p = 0,0004).

We also identified in *P*. *rhodozyma* one pair of divergently transcribed HD-like genes (Fig 3), which are usually hyperpolymorphic in heterothallic, tetrapolar basidiomycetes [8]. The HD-like genes were located in a scaffold different from the one harboring the *P/R* clusters in the three available draft genome assemblies, the two loci being therefore probably genetically unlinked. A comparison of the complete protein sequences encoded by representatives of all four *P*. *rhodozyma* populations shows that the proteins are quite similar among different strains (S2 Fig). Similarly to what was observed for the *P/R* loci, we found that *HD1* and *HD2* gene sequences retrieved from strains representing all four *P*. *rhodozyma* populations recapitulate previously reported phylogenetic relationships between populations (S3 Fig). Interestingly, the homeodomain region of the HD2 protein is strictly conserved in all the variants identified, while variable amino acid positions seem to be evenly distributed throughout the remainder of the protein (S2 Fig). This is in contrast to what is usually observed for different alleles of *HD2* in tetrapolar species, where the N-terminal dimerization domain is much more divergent than the C-terminal domain, as a result of negative frequency dependent selection imposed by the functional constraints on the N-terminus of both the HD1 and HD2 proteins [49]. Notably, HD2 variants representing different *P*. *rhodozyma* populations exhibited different C-terminal domain lengths, except for the shortest variant (S2 Fig) that was found in strains representing both populations B and C. A similar analysis conducted for HD1 protein sequences revealed one conservative amino acid substitution within the homeodomain, identical lengths for all the variants and an even distribution of polymorphic sites, as observed for HD2 (S2 Fig).

In summary, we conclude that no evidence could be found for the existence of polymorphisms at the *MAT* loci that might represent cryptic molecular mating types in a set of *P*. *rhodozyma* strains that captures the diversity found so far within the species.

### One pheromone receptor is necessary and sufficient for sexual reproduction

In heterothallic systems, pheromones and pheromone receptors are usually involved in the process of cell-cell compatibility recognition preceding plasmogamy [1,13]. This process might conceivably be dispensable in self-fertile sexual reproduction but was, on the contrary, found to be relevant for homothallic systems in species of the genera *Neurospora* [50] and *Sordaria* [51] (Ascomycota). To address the question of whether pheromone receptor genes were required for sexual reproduction of *P*. *rhodozyma*, we produced deletion mutants of each of the two receptor genes *STE3-1* and *STE3-2* in turn, using homologous recombination to target chromosomal integration of antibiotic resistance markers to the *P/R* locus so as to delete each of the receptor genes, but leaving the pheromone precursor genes intact (Fig 1 and Table 1). Assessment of the phenotype of the individual pheromone receptor mutants, *ste3-1Δ* and *ste3-2Δ*, in sporulation medium showed that their sporulation capabilities were similar to the wild type suggesting that neither receptor is required *per se* for sporulation (Fig 1E). On the contrary, the double mutant, *ste3-1Δ ste3-2Δ*, lacking both receptors, failed completely to sporulate indicating that the two receptors are functional and redundant. We subsequently reintroduced the *STE3-1* gene in the double mutant *ste3-1Δ ste3-2Δ*, through integration in the rDNA locus. As expected, functional complementation restored the capability of the resulting strain *ste3-1Δ ste3-2Δ* +*STE3-1* to sporulate (Fig 1C).

**Table 1:** List of *P*. *rhodozyma* strains.

Strains / Mutants	Relevant genotypes
*CBS 6938*	*Phaffia rhodozyma*
*ste3-1Δ*	*ste3-1Δ*::*G418*^*1*^
*ste3-2Δ*	*ste3-2Δ*::*HYG*^*2*^
*ste3-1Δste3-2Δ*	*ste3-2Δ*::*HYG*^*2*^*/ste3-1Δ*::*G418*^*1*^
*hd1Δ*	*hd1Δ*::*HYG*^*2*^
*hd2Δ*	*hd2Δ*::*G418*^*1*^
*hd1Δhd2Δ*	*hd1Δ*::*HYG*^*2*^*/hd2Δ*::*G418*^*1*^
*ste3-1Δmfa1Δ*	*ste3-1/mfa1Δ*::*G418*^*1*^
*ste3-2Δmfa2Δ*	*ste3-2/mfa2Δ*::*G418*^*1*^
*ste3-1Δmfa2Δ*	*ste3-1Δ*::*G418*^*1*^*/mfa2Δ*::*ZEO*^*3*^
*ste3-2Δmfa1Δ*	*ste3-2Δ*::*HYG*^*2*^*/ mfa1Δ*::*ZEO*^*3*^
*ste3-1Δmfa1Δhd1Δ*	*ste3-1/mfa1Δ*::*G418*^*1*^*/hd1Δ*::*HYG*^*2*^
*ste3-2Δmfa2Δhd2Δ*	*ste3-2/mfa2Δ*::*G418*^*1*^*/hd2Δ*::*ZEO*^*3*^
*spo11Δ*	*spo11Δ*::*HYG*^*2*^
*ste3-1Δste3-2Δ+STE3-1*	*ste3-2Δ*::*HYG*^*2*^*/ste3-1Δ*::*G418*^*1*^*/STE3-1*::*ZEO*^*3*^*+ rDNA*
*ste3-2Δmfa2Δ+MFA2*	*ste3-2/mfa2Δ*::*G418*^*1*^*/MFA2*::*ZEO*^*3*^*+ rDNA*
*hd1Δ+HD1*	*hd1Δ*::*HYG*^*2*^*/HD1*::*ZEO*^*3*^*+ rDNA*
*hd1Δhd2Δ+HD1*	*hd1Δ*::*HYG*^*2*^*/hd2Δ*::*G418*^*1*^*/HD1*::*ZEO*^*3*^*+ rDNA*

^1^ Geneticin resistance cassette

^2^ Hygromycin resistance cassette and

^3^ Zeocin resistance cassette.

### Evidence for reciprocal compatibility between receptors and pheromones encoded by the two clusters

Genes encoding distinct pheromone precursors (*MFA1* and *MFA2*) flank each of the two pheromone receptor genes (*STE3-1* and *STE3-2*) that are located at a short distance from each other in the genome (Fig 1A). This arrangement is reminiscent of the *P/R* locus model proposed for the mushroom *Coprinopsis cinerea*, a tetrapolar species also possessing multiple *P/R* clusters per individual [42]. Should the *P*. *rhodozyma P/R* clusters functionally resemble those of *C*. *cinerea*, the receptor would be expected to be insensitive to the pheromone encoded by its flanking gene. To find out how, if at all, the pheromones encoded in the *P*. *rhodozyma P/R* clusters interact with the receptors, we constructed two deletion mutants, *ste3-1Δmfa1* and *ste3-2Δmfa2*. Neither of the resulting strains was able to sporulate, strongly suggesting that each receptor is activated by the pheromone encoded in the other cluster, i.e. Ste3-1 is for example very likely activated by Mfa2 (Fig 1C). In accordance with this, reintroduction of the *MFA2* gene in the *ste3-2Δ mfa2Δ* mutant complemented its sporulation defect (Fig 1C). We subsequently constructed double deletion mutants *ste3-1Δmfa2Δ* and *ste3-2Δmfa1Δ*, each expressing a distinct, presumably interacting pheromone receptor/pheromone pair. Both double mutants were able to sporulate at normal levels (Fig 1C and 1F) showing that, as predicted, Mfa1 interacts with Ste3-2 while Mfa2 activates Ste3-1.

Finally, when the *ste3-1Δ mfa1Δ* and *ste3-2Δ mfa2Δ* mutants are co-cultured in suitable medium, a low level of sporulation is observed (Fig 1 and S2 Table), two orders of magnitude lower than wild type, which is consistent with the functional receptor remaining in each strain being activated by the pheromone produced and secreted by the other strain.

### HD1 and HD2 are required for sexual development in *P*. *rhodozyma*

Divergently transcribed candidate *HD1* and *HD2* genes were also identified in the *P*. *rhodozyma* genomes, resembling the genomic arrangement of homologous genes found in most tetrapolar species (Fig 3). Hence, we proceeded to investigate whether the putative *HD1* and *HD2* genes uncovered in the genomes were involved in sexual development. To that end, single mutants, *hd1Δ* and *hd2Δ*, in which each of the two genes was deleted in turn, as well as a double mutant *hd1Δhd2Δ*, were constructed. Sporulation was abolished in the *hd1Δ* single mutant and in the *hd1Δ hd2Δ* double mutant, but vestigial sporulation was observed for the *hd2Δ* mutant (Fig 3 and S3 Table). Reintroduction of the *HD1* gene in the rDNA of the *hd1Δ* strain restored the wild type sporulation phenotype, confirming that loss of sporulation was truly a consequence of *HD1* deletion (Fig 3C and 3D). Similarly, ectopic expression of *HD1* in the *hd1Δ hd2Δ* mutant restored a limited ability to sporulate to levels similar to those observed for the *hd2Δ* mutant (S3 Table). Taken together, these results suggest that sporulation requires both proteins but that the absence of HD2 does not completely block completion of the sexual cycle. The most likely explanation for these observations is that the divergently transcribed *HD1* and *HD2* genes in *P*. *rhodozyma* work together to regulate genes required for sexual development, unlike similarly arranged genes found in tetrapolar species across the Basidiomycota. To assess whether the HD1 and HD2 proteins might be capable of forming a heterodimer, we first used a bacterial two-hybrid assay [52] to try to detect an interaction between the HD1 and HD2 proteins of *P*. *rhodozyma*. The results, shown in S4 Fig, fail to consubstantiate the occurrence of an interaction between the two proteins sufficiently strong to be detected by this assay. To consubstantiate this apparent absence of interaction, we subsequently performed a second assay using the yeast two-hybrid system, which was previously used to detect interactions between HD1 and HD2 proteins of *U*. *maydis* [14] and *C*. *neoformans* [53]. To this end, fusions were constructed mimicking those successfully employed to detect interactions between the *U*. *maydis* proteins, including fusion proteins comprehending the complete HD proteins as well as shorter versions including solely the N-terminal domains normally involved in dimerization and the homeodomain region (S4 Table). In line with results obtained for the bacterial two-hybrid system, we did not detect a clear interaction using the two possible combinations of the short dimerization domains of HD1 and HD2 (S5 Fig) with only one of the four transformants expressing the HD1 and HD2 N-terminal domains showing some activation of the *MEL1* reporter gene and no discernible activation of the remaining two reporter genes. However, a weak interaction signal denoted only by the *MEL1* reporter gene was consistently detected in all combinations involving fusion proteins that comprehended one complete coding region of either HD1 or HD2 (S5 Fig). Taken together, these results lead to the tentative conclusion that the two *P*. *rhodozyma* proteins may interact, albeit weakly. Interestingly, in the yeast assay, we also observed interactions that might support the formation of homodimers (S5 Fig). Homodimers of homeodomain proteins were observed in *S*. *cerevisiae* where they have a well-defined function [54], but no biological roles have been ascribed so far to HD protein homodimers in the basidiomycetes.

### Genetic evidence for the involvement of meiosis in the sexual cycle of *P*. *rhodozyma*

**Fig 4:**
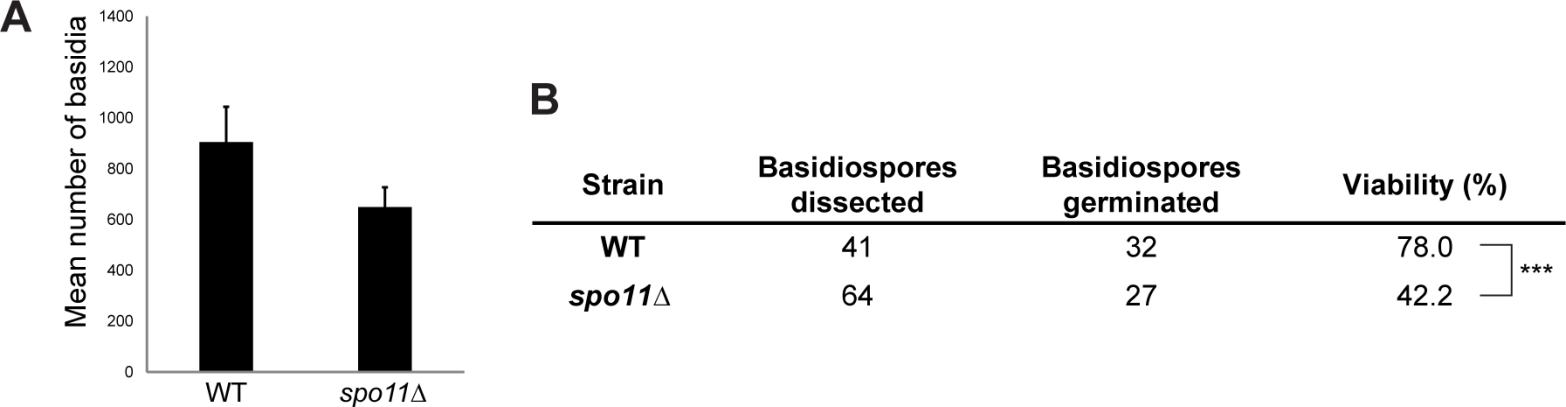
SPO11 deletion mutant. **A.** Number of basidia observed per plate in sporulation proficient mutants after 10 days of incubation in sporulation medium at 18°C. Error bars represent standard deviations from the mean for the three biological replicates. No significant differences were observed between the sporulation ability of the wt and *spo11Δ* mutant. **B.** Total number of basidiospores recovered from wt and *spo11Δ* mutant and fraction of viable spores (asterisks denote significant difference, P value = 0.000302, *Chi-square* statistic).

Close inspection of the *P*. *rhodozyma* genomes available revealed the presence of all but one (*MLH2)* of the core genes required to complete meiosis [39] (Nicolás Bellora and Diego Libkind, personal communication). However, some available evidence also argued against the occurrence of a typical meiosis: the ploidy of different *P*. *rhodozyma* strains is uncertain and may vary [40], extensive intraspecies chromosome length polymorphisms were noted [36,55,56] and segregation of markers was observed to deviate from Mendelian distribution [36]. This prompted us to examine the dependence of *P*. *rhodozyma* sporulation on *SPO11*, a core meiosis gene encoding an endonuclease [57] shown to be required for meiotic recombination in *C*. *neoformans* [58]. To achieve that, we compared the ability of a *spo11Δ* mutant to complete the sexual cycle and the viability of the spores produced with that of the wild type strain. The results, shown in Fig 4, indicate that sporulation is less efficient in the *spo11Δ* mutant than in the wild type, although this difference did not reach statistical significance. In addition, the viability of F1 spores isolated from the *spo11Δ* mutant was significantly lower than that of the wild type. These results suggest that meiosis is indeed part of the sexual life cycle of *P*. *rhodozyma*.

### Construction of heterothallic strains

**Fig 5:**
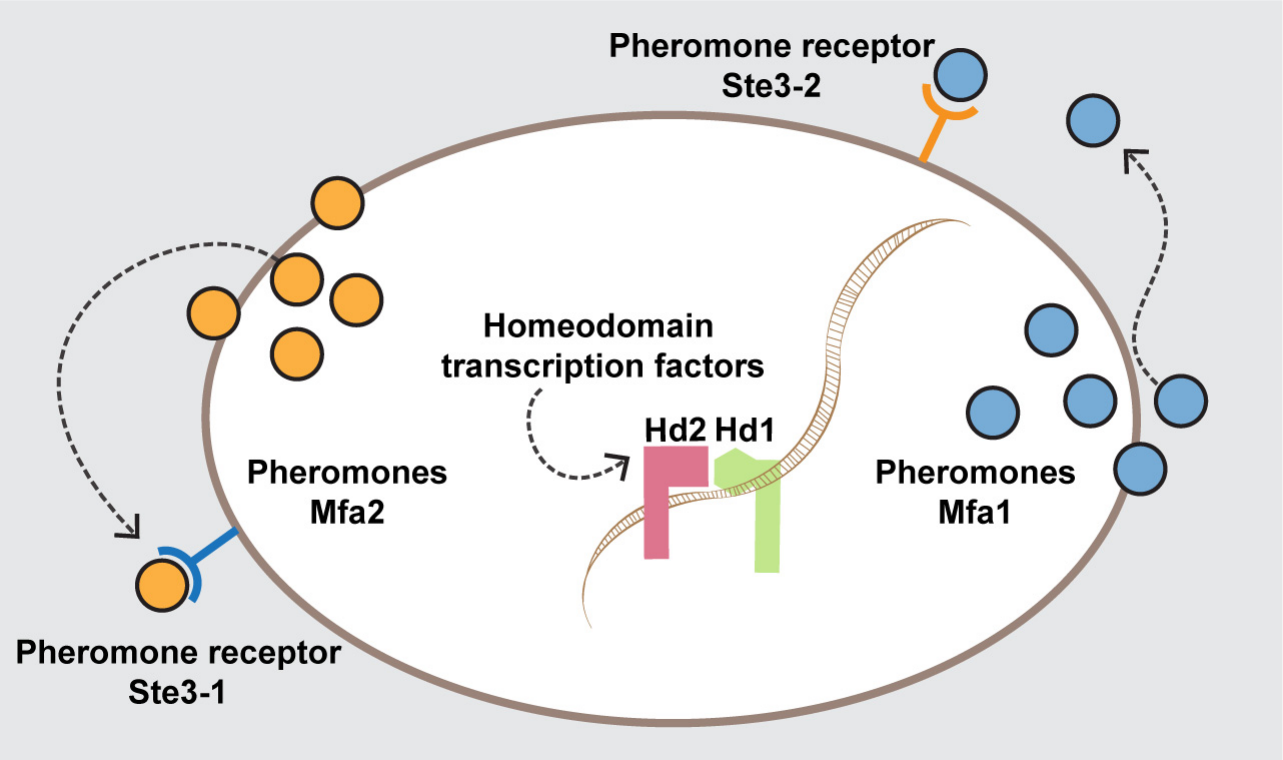
Model describing molecular interactions required for sexual reproduction. Pheromones and receptors encoded in the *P/R* locus were both shown to be reciprocally compatible, forming two functionally redundant pairs. The HD1 and HD2 proteins encoded at the *HD* locus are proposed to contribute synergistically for sexual development, possibly through a weak interaction allowing them to form a stable heterodimer upon DNA binding.

Interestingly, the results described in the previous sections show that the genetic determinants and mechanisms involved in the homothallic life cycle of *P*. *rhodozyma* are similar to those of heterothallic, tetrapolar basidiomycetes. On the other hand, from the point of view of strain improvement of *P*. *rhodozyma* for biotechnological applications, it would be very useful to be able to generate strains in which outcrossing is strongly favored, because it facilitates the selection of strains harboring desirable combinations of characteristics using classical genetic approaches. This prompted us to use our recently acquired knowledge of the sexual reproduction system to try to generate obligate outcrossing *P*. *rhodozyma* strains. To do that, we generated artificial “mating types” by constructing two triple mutants harboring complementary components of the two mating recognition systems, *ste3-1Δ mfa1Δ hd1Δ* and *ste3-2Δ mfa2Δ hd2Δ*. Our model (Fig 5) predicts that a cross between these two triple mutants should almost exclusively yield spores resulting from conjugation between independent cells belonging to complementary “mating types”, while formation of basidia originating from single cells or from pedogamy is almost completely prevented by the absence of complete *HD1/HD2* gene pair in each of the “mating types”. Hence, while in a cross between *ste3-1Δ mfa1Δ* and *ste3-2Δ mfa2Δ* mutants, extracellular diffusion of the pheromones would permit any of the three possible modes for formation of basidia (S1 Fig) because each mutant possessed a complete *HD1/HD2* pair, in the triple mutant cross, sporulation is expected to occur almost exclusively after cell fusion brings together the HD1 and HD2 partners originating from different “mating types”. Indeed, in a cross between the artificially created “mating types”, basidia with basidiospores were formed, albeit at levels three orders of magnitude lower than wild type and two orders of magnitude lower than the cross between *ste3-1Δ mfa1Δ* and *ste3-2Δ mfa2Δ* (S2 Table). The occurrence of sporulation in the triple mutant cross, even if at very low levels, paves the way for strain improvement based on outcrossing.

## Discussion

Here we characterize in detail the *MAT* loci of *P*. *rhodozyma*, an attractive model in which to study homothallism in basidiomycetes due to its genetic amenability and to the availability of draft genome sequences [39] (Nicolás Bellora and Diego Libkind, personal communication). In line with previous observations, all strains we examined are homothallic, which agrees with the limited role previously proposed for sexual recombination and hybridization between natural *P*. *rhodozyma* populations [35]. Our survey of variants of *MAT* genes in strains representing the four natural populations identified so far in *P*. *rhodozyma* failed to uncover additional potentially functionally divergent homologs of *MAT* genes that could represent cryptic molecular mating types. Indeed, the presence in a single genome of two sets of genes encoding pheromone precursors and pheromone receptors could represent a heterothallic *MAT* locus resembling that of *C*. *cinerea* where each mating type may harbor several receptor and pheromone precursor genes [9,42]. However, analysis of *MAT* gene sequences identified in *P*. *rhodozyma* strains do not favor this possibility, because they were all very similar in the different strains examined (S3 Fig). Therefore, the present study suggests that the species may be considered exclusively homothallic in the sense that no molecular mating types were identifiable, although it admits both uniparental and biparental (outcrossing) modes, the first being apparently much more frequent across a set of strains representing diversity within the species, as supported also by population analysis [35].

Primary homothallism has been observed in a considerable number of species across the entire fungal kingdom [18,19], sometimes in a few strains of an otherwise heterothallic species [11,43] or, alternatively in lineages consisting mainly of species formed entirely of homothallic individuals with a few heterothallic species, as observed in Aspergilli [19]. In a number of cases, like for *A*. *nidulans*, it was possible to demonstrate that homothallism was associated to the presence in one individual of the entire complement of genetic information normally present in the two opposite mating types of heterothallic individuals of closely related species [22]. Also, the purposeful introduction of genes determining sexual identity in one mating type into the opposite mating type, resulted in the emergence of a homothallic phenotype [59]. Nevertheless, a thorough explanation of how these genes interact to produce the homothallic phenotype is still lacking in most cases [1,18,19,28]. The genetic makeup of *MAT* loci in *P*. *rhodozyma*, although suggestive of primary homothallism, departs in important aspects from what would be expected from a simple assemblage in a single genome of two mating types as typically found in the Agaricomycotina. Firstly, in sexually reproducing species most closely related to *P*. *rhodozyma*, two lineages of pheromone receptors can be clearly discerned exhibiting trans-specific polymorphism [9], while our phylogenetic analysis shows with a high degree of confidence that the two receptors in *P*. *rhodozyma* are more closely related with each other than with receptors in other species. Therefore, our results strongly suggest that *P*. *rhodozyma* receptors, possibly similarly to species in the Agaricales (mushrooms), may descend from only one of these lineages [9]. A consequence of this is that the two pheromone/receptor pairs seem to have diverged relatively recently, at least after separation of the *Phaffia* and *Cryptococcus* lineages. Both receptors are functional and are activated by the pheromone encoded in the other cluster. We posit that the most likely setting for these pheromone/receptor specificities to have evolved is a heterothallic system, which supports that an ancestor of the *Phaffia* lineage was likely heterothallic.

Primary homothallism is thought to be rather uncommon in Basidiomycetes, which is in line with the complex genetic underpinnings of the mating system in this phylum, involving two independent non-self recognition checkpoints. Hence, a transition from heterothallism to primary homothallism would require a genomic rearrangement gathering in the same genome two compatible versions of both the *P/R* and *HD* loci, or that one or both loci would become self-compatible. It seems more likely that homothallism in *P*. *rhodozyma* is derived from a tetrapolar ancestor, rather than bipolar, because the *HD* and *P/R* loci are apparently genetically unlinked [39] (Nicolás Bellora and Diego Libkind, personal communication) and are both required for sporulation. However, the extant *MAT* locus in *P*. *rhodozyma* is not simply a gathering of all *MAT* genes (*P/R* and *HD*) normally present in two compatible mating types in tetrapolar systems because only one *HD* gene pair is present, encoding proteins that are both required to promote sexual development, as shown by the absence of sporulation in the *hd1Δ* and by a dramatic drop in sporulation (approximately 0.2% of wild type sporulation levels remaining) observed for the *hd2Δ* mutant. In heterothallic systems, HD proteins form heterodimers in which the interacting partners are encoded by different mating types, thereby enforcing mating between genetically distinct individuals (outcrossing). The domain structures of HD proteins and the molecular interactions they are likely to undergo have been examined in detail in *U*. *maydis* [14,15,60,61], *C*. *cinerea* [62], *Schizophylum commune* [63] and *C*. *neoformans* [53]. These studies showed that the protein domains required for proper functioning of the heterodimeric transcription factor, such as high affinity DNA binding, nuclear localization signal (NLS) and transcriptional activation were not present in a single protein. For example, the *C*. *cinerea* HD1 protein harbors a transcriptional activation domain and a NLS but its homeodomain is dispensable for sexual development, while its HD2 counterpart possesses no NLS but its homeodomain is absolutely required for DNA binding by the heterodimer [64]. In this manner, undimerized partners are doomed to be unsuccessful as transcription factors: HD2 is unable to get transported into the nucleus on its own while HD1 can get transported into the nucleus due to its NLS, but will fail to effectively bind DNA [64]. Conversely, in the HD2 proteins of *Heterobasidion*, no *bona fide* homeodomain could be found, so that DNA binding probably relies entirely on the homeodomain of the HD1 protein [65]. Interestingly, in *P*. *rhodozyma* we detected at most a weak interaction between the two HD proteins. This is in contrast to the results obtained when *U*. *maydis* [14] and *C*. *neoformans* [53] HD proteins were examined in a yeast two-hybrid assay. However, phenotypes of the various deletion mutants showed clearly that HD1 and HD2 are involved in the regulation of an overlapping set of genes essential for sporulation. Taking into account the well-established mode of operation of model species within the Agaricomycotina, this leaves room for two possible interpretations: i) the two *P*. *rhodozyma* HD proteins do not form heterodimers, as normally observed for proteins encoded by the same locus, and hence, they have independent contributions to regulate a set of genes essential for sexual development; ii) the two proteins interact to promote transcriptional regulation of genes essential for sporulation, but the interaction is much weaker than those previously characterized in heterothallic species. We favor the latter possibility for a number of reasons. Firstly, it has been shown for mutant alleles in *U*. *maydis* that they can promote sexual development *in vivo* despite their failure to interact detectably in the yeast two-hybrid assay [14]. Secondly, we found that HD1 alone supports the ability to sporulate, albeit at low levels (Fig 3 and S3 Table). This is consistent with, for example, a scenario in which HD1 is dependent on HD2 for high affinity DNA-binding, but can bind independently sufficiently well to support a low level of sporulation in the absence of the HD2 partner. In line with this, a prominent DNA binding role has been ascribed to the HD2 proteins of other members of the Agaricomycotina, such as *C*. *cinerea* [64], *S*. *commune* [66] and *C*. *neoformans* [67].

The third reason in favor of a weak interaction between the two HD proteins pertains to how self-compatibility of the *P*. *rhodozyma* HD protein pair, if it exists, may have evolved. For this, two possibilities may be considered. Firstly, a *HD* gene pair encoding self-compatible proteins could conceivably form through recombination between two distinct heterothallic ancestor alleles, in which case the interaction between the two proteins would probably be expected to be sufficiently strong to be unequivocally detected in the two hybrid assays, as observed for the large majority of naturally occurring *HD* alleles in the heterothallic species examined [14,53]. However, in face of previous findings [14], a very likely second evolutionary path to generate a compatible HD1/HD2 pair would be the emergence of one or more mutations relieving the structural hindrance normally preventing interaction between HD proteins encoded by the same locus. It has been previously shown that a single amino acid mutation may be sufficient to remove the obstacle for self-dimerization [14]. In that case, a weak interaction permitting for example cooperative DNA binding would probably suffice for normal function provided the two proteins can reach the nucleus independently. In fact, HD1 is apparently able to reach the nucleus independently of HD2, because it is capable of promoting some sporulation in the absence of HD2, although inspection of the sequences of both proteins revealed that only HD2 possessed a candidate NLS (S2 Fig). However, and in the absence of an unequivocal experimental demonstration of an interaction, the establishment of a definite mode of action for the HD proteins will have to await a detailed dissection of the functional domains present in each of the two proteins and the identification of their DNA binding sites, as previously accomplished in other systems [14,15,49,53, 60, 61, 66, 67].

While contemplating the various possibilities to reconcile the lack of a strong interaction between the HD1 and HD2 proteins of *P*. *rhodozyma* with the phenotypes exhibited by the various mutants, we also considered the possibility that HD1 might engage an alternative dimerization partner in the absence of HD2. However, close inspection of all *P*. *rhodozyma* genomes available, failed to detect genes encoding homeodomain proteins with the appropriate domain architecture (S5 Table). Hence, we consider this possibility very unlikely.

The genetic arrangement of the two P/R clusters is strongly reminiscent of P/R clusters in many basidiomycetes, each functional receptor gene being in the vicinity of a gene encoding a functional pheromone that nevertheless fails to activate its receptor counterpart in the same cluster or locus [8,42]. It can therefore be easily envisaged that this arrangement resulted from the fusion of two heterothallic loci.

We propose a model (Fig 5) summarizing the most likely roles of the six *MAT* genes in the sexual cycle of *P*. *rhodozyma* given our present knowledge concerning the genetics of *MAT* in *P*. *rhodozyma* and taking into account available information for other species in the Agaricomycotina. With this model as starting point, we predicted that it would be possible to generate artificial heterothallic “mating types” in *P*. *rhodozyma*, by constructing strains containing one *P/R* cluster and one *HD* gene complementary to those present in the other “mating type”. This was confirmed in successful heterothallic crosses between the *ste3-1Δ mfa1Δ hd1Δ* and *ste3-2Δ mfa2Δ hd2Δ*. The possibility of outcrossing in *P*. *rhodozyma* had been previously put forward based on the observation of fusion between independent cells and on genetic evidence, including strains that seem to be hybrids between the known *P*. *rhodozyma* populations [35,36]. However, in line with previous observations [35], our results indicate that outcrossing is probably infrequent, since sporulation is greatly diminished when the possibility of selfing is genetically prevented (S2 Table). The triple mutant crosses open a new avenue to facilitate improvement of *P*. *rhodozyma* strains for astaxanthin production, for example by combining industrially attractive features like growth at higher temperatures with genetic alterations that improve astaxanthin production.

We also gathered evidence that *P*. *rhodozyma* probably undergoes meiosis during its life cycle since deletion of a core meiosis gene shown to be required for meiosis in *C*. *neoformans* [58] the model organism most closely related to *P*. *rhodozyma*, possibly affects sporulation efficiency and significantly decreased spore viability. We deem it therefore unlikely that in *P*. *rhodozyma* ploidy changes are achieved by distinct mechanisms as postulated to occur in the parasexual cycles of other fungi, like the yeast *C*. *albicans* [68].

*P*. *rhodozyma* belongs to a lineage that corresponds to the order Cystofilobasidiales, comprising species consisting entirely of homothallic individuals and others that are heterothallic [69]. Homothallism has been suggested to be an evolutionary dead end so that transitions are proposed to be unidirectional, from heterothallic to homothallic [70,71]. The genetic makeup of the *P*. *rhodozyma* mating system presently described is in line with this hypothesis, since it suggests a transition from a (tetrapolar) heterothallic ancestor to homothallism. It will be very interesting to find out whether transitions in the entire clade of the Cystofilobasidiales also conform to this unidirectionality. To investigate that and other aspects of the biology of sexual reproduction in this group of organisms, we are currently using comparative genomics to illuminate the molecular underpinnings of the various mating systems in the Cystofilobasidiales. Evolution of *HD* compatible gene pairs and evolutionary mechanisms associated with transitions between homothallism and heterothallism will be addressed.

## Materials and Methods

### Strains and culture conditions

*Escherichia coli* DH5α (S6 Table) strain was used for all cloning steps and was grown in LB medium (10 g/L Tryptone, 5 g/L Yeast Extract and 5 g/L NaCl) with 100 μg/ml ampicillin at 37°C. Wild type *Phaffia rhodozyma* strain CBS 6938 was grown in YPD medium (10 g/L Yeast Extract, 20 g/L Peptone and 20 g/L glucose) at 20°C, while mutants strains were grown in YPD medium supplemented with appropriate antifungal drugs (50 μg/ml geneticin, 50 μg/ml hygromycin or 100 μg/ml zeocin). All wild type *P*. *rhodozyma* and mutant strains used and generated in this work are listed in Table 1.

### Construction of the gene deletion fragments and complementation plasmids

Different gene deletion fragments (GDF) were generated in order to construct *P*. *rhodozyma* deletion mutants, using a common strategy, consisting in cloning the upstream and downstream flanking regions of the selected gene upstream and downstream of an antifungal resistance cassette, to promote integration of the GDF in the targeted genomic locus [34]. Standard molecular biology methods were employed [72,73] and three distinct plasmids were used as backbone, namely pPR2TN [74], pBS-HYG [75] and pJET1.2+ZEO (S7 and S8 Tables), encoding geneticin, hygromycin and zeocin resistance genes, respectively (S6 Fig). Specific primers for the *STE3-1*, *HD1*, *HD2*, *SPO11*, regions *STE3-1/MFA1* and *STE3-2/MFA2* flanking regions were designed to include restriction sites that allowed cloning of the amplified regions onto the chosen plasmids (S7 Table). The GDF for the *STE3-2* gene was constructed by overlap extension PCR and then cloned into the pJET1.2 vector using CloneJET PCR Cloning Kit (Thermo Scientific) (S8 Table). Nested primers were used to amplify the complete GDFs by PCR using Phusion High-Fidelity DNA Polymerase (Thermo Scientific) (S7 and S8 Tables). PCR products were purified using GeneJET Gel Extraction Kit (Thermo Scientific) or Illustra GFX PCR DNA and Gel Band Purification Kit (GE Healthcare) and finally used to transform *P*. *rhodozyma*. Complementation of selected deletion mutants was accomplished using plasmid pUC18+rDNA+ZEO that was constructed by inserting the constructed 1.8 Kb zeocin resistance cassette (S9 Table) and a 3 Kb fragment of the rDNA from plasmid pPR2TN in plasmid pUC18 at the SmaI and SacI restriction sites respectively. DNA fragments containing the ORFs’ of the genes pertaining to the complementation with approximately 300 bp flanking regions, were subsequently cloned into the PstI and BamHI restriction sites of pUC18+rDNA+ZEO. Each of the four complementation plasmids were then linearized with ClaI within the rDNA sequence to promote integration of the plasmid into the ribosomal DNA of the mutant strains to be complemented (S10 Table) [76].

### Transformation of *Phaffia* strains and mutant confirmation

Linearized plasmids and GDF were used to transform *P*. *rhodozyma* by electroporation as previously described (S7–S10 Tables) [76]. Transformants were selected in YPD medium with the appropriate antifungal drugs. Mutants with multiple deletions were obtained by transforming a confirmed deletion mutant with a second or third GDF. Correct integration of the disruption cassettes was verified by PCR (S7–S10 Tables) as previously described [34]. Briefly, a primer inside the resistance cassette and a primer outside of the flanking region present in the GDF at both the 5’ and 3’ extremities were used in diagnostic PCR reactions to identify the desired mutants. Absence of the gene targeted for deletion was also assessed by PCR for each mutant. Key deletion mutants were also confirmed by Southern blot in order to ensure that integration of the GDF occurred only once and in the correct locus. For Southern blot, 5μg of genomic DNA was digested with ClaI and run in a 0.8% agarose gel. Southern blot was performed using standard methods. Primers MP091-MP092 and MP062-MP063 were employed to amplify fragments of the resistance genes present in the geneticin and hygromycin cassettes to be used as probes. Labeling of the probes was performed with (α-^32^P) dATP using the Prime-a-Gene Labelling system (Promega). Signals were detected on X-ray films (Hyperfilm MP, GE Heathcare Life Sciences) (S7 Fig).

### Sporulation assays

In order to test the ability of the deletion mutants to sporulate, the various mutants were inoculated on DWR (2.5% agar and 0.5% ribitol) solid medium, incubated at 18°C and observed regularly for up to two months. Sporulation efficiency assays were performed as described previously by Kucsera [36]. Briefly, cells were grown on YPD medium overnight (180 rpms, 20°C, in 10% of the volume of the flask), collected by centrifugation and washed thoroughly with sterile distilled water to remove culture medium. Cells were subsequently distributed in 10 μl drops over the surface of DWR plates that were subsequently incubated at 18°C for 10 days. The number of basidia on each plate was determined by direct observation of the perimeter of the colonies using an optical microscope. Three independent assays were performed with CBS 6938 wild type (WT) strain and with all deletion mutants (in triplicate). Student’s t-test was performed (with a significance of 99%) to ascertain the statistical significance of the differences observed between the WT and each of the sporulating deletion mutants. Additional assays were conducted to determine the viability of F1 progeny of the *spo11Δ* mutant and wild type strain CBS 6938. Basidiospores were recovered by micromanipulation and were transferred to YPD solid medium to determine viability. Chi-square statistic was performed to verify if the difference between WT and *spo11* deletion mutant was statistically significant.

### Crosses of mutant strains

Mutant strains to be crossed were cultivated and washed as previously described, were mixed 1:1 and were finally distributed in 10 μl drops on the surface of DWR plates. Additionally, direct mixture of strains on DWR plates was also performed. Plates were incubated at 18°C and observed daily.

### Bacterial two-hybrid assays

In order to ascertain the possible interaction between HD1 and HD2 protein bacterial two-hybrid assays were performed using the Bacterial adenylate cyclase two-hybrid system kit from Euromedex. The cDNA of each of the *HD* genes (S1 File) was synthesized at Eurofins and delivered as an insert in vector (pEX-K4). Synthetic *HD* genes were amplified by PCR (S11 Table) and sub-cloned into plasmids pKNT25 and pUT18 using the *Hind III* and *Pst I* restriction sites present in the MCS of both of plasmids, according to standard molecular biology techniques and the Euromedex manual [52]. Genes were cloned in frame at the N-terminus end of the T25 and T18 peptides. After transformation, integrity of the constructs was assessed by PCR amplification and sequencing of the cloned fragments (primers MP191/MP192 and MP193/MP194) (S11 Table). Different combinations of the recombinant plasmids (S4C Fig) were co-transformed into BTH101 *E*. *coli* cells (S6 Table). After successful transformation, the phenotype of 8 clones (identified as 1.1–10.8) of each of the different co-transformations was assessed in X-gal and MacConkey/maltose media upon incubation at 30°C for 48h. Results were scored after 24h and 48h incubation times. The presence of both fragments and correct plasmids in each of the clones was assessed by PCR (S11 Table).

### Yeast two-hybrid assays

Matchmaker Gold Yeast Two-Hybrid System from Clontech was also used to assess a possible interaction between the homeodomain proteins of *P*. *rhodozyma*. Synthetic HD genes (S1 File) were cloned into pGBKT7 and pGADT7 plasmids by transforming each of the PCR fragments and the digested plasmid into the pertinent yeast strain (S4 Table and S5C Fig). All *S*. *cerevisiae* transformations were performed according to Yeastmaker Yeast Transformation System 2 User Manual from Clontech. Primers used to amplify the *HD* genes carried 40 bp 5’ tails homologous to the ends of the linearized plasmids to promote recombination. Plasmids pGBKT7 and pGADT7 were linearized with *Pst I* and *Cla I*, respectively. Two distinct versions of the *HD* genes were used, a shorter one comprising the complete N-terminal and homeodomain region of each of the genes (the first 183 amino acids of HD1 protein and the first 196 amino acids of HD2 protein respectively) and another, comprising the complete proteins. Each of the haploid *S*. *cerevisiae* strains generated was tested for the ability of the fusion protein expressed to activate any of the reporter genes on their own (S5E Fig). Haploid *S*. *cerevisiae* strains (S5D Fig) were them mated and diploid strains selected as described in the Matchmaker Gold Yeast Two-Hybrid System user manual. Three diploid strains (named X, Y and Z), selected from each mating experiment were tested for their ability to activate the reporter genes. Plates were incubated at 30°C for 72h and photographed daily (S5 Fig).

### Construction of Ste3 phylogeny

Previously published pheromone receptor sequences were used to reconstruct the phylogenetic tree depicted in Fig 2. The accession numbers of sequences used are listed in S12 Table. Available draft genome sequences of *Kwoniella mangrovensis* CBS 10435, *K*. *heveanensis* BCC 8398 and *Tremella fuciformis* tr26 were searched for the presence of pheromone receptor homologues by TBLASTN using *P*. *rhodozyma* Ste3-1 as query. Genomic regions corresponding to positive hits were retrieved (GenBank accession numbers ASQD01000019.1, ASQB01000005 and LBGW01000351, respectively) and protein sequences were deduced after removal of likely introns, either manually or using AUGUSTUS [77]. The final protein dataset was aligned using an iterative refinement method (L-INS-i) in MAFFT v.7.221 [78]. Poorly aligned regions were removed with trimAl v.1.2 [79] using the "gappyout" option. The resulting alignment containing 338 positions was analyzed in ProtTest v.3.2 using the corrected Akaike information criterion (AICc) to determine the model of sequence evolution that best fitted our data. A maximum likelihood-based phylogenetic tree was built in RAxML v.8.1.24 using PROTGAMMAILGF model of amino acid substitutions and branch support was determined using 1000 rapid bootstraps. The *Saccharomyces cerevisiae* Ste3 pheromone receptor was used to root the tree.

### Construction of *MAT* phylogenies

*MAT* genes *STE3-1*, *STE3-2*, *HD1* and *HD2* were partially amplified and sequenced using primers listed on S7 and S8 Tables. All sequences obtained were deposited in Genbank and are listed in S12 Table. Nucleotide unrooted maximum likelihood phylogenies were inferred with General Time Reversible model and 1000 bootstrap replications on MEGA5.1 software [80]. The trees with the highest log likelihood are shown with branch lengths measured in the number of substitutions per site.

## Supporting Information

S1 Fig *P*. *rhodozyma* life cycle. Vegetative cells propagate by budding. Nitrogen depletion and the presence of polyols trigger the formation of an aerial basidium that gives rise to apical basidiospores. Three distinct cellular events were observed to give rise to formation of the basidium: conjugation of independent cells, conjugation between mother-cell and bud (pedogamy) and single cells.
(PDF)

S2 Fig Protein alignments of proteins Ste3-1, Ste3-2, Ste3-1 vs Ste3-2, Mfa1, Mfa2, Hd1 and Hd2. Sequences were retrieved from the available genomes of *P*. *rhodozyma* strains (CBS 7918, CBS 6938 and CRUB 1149) and obtained through this work (S12 Table). Alignment was performed using ClustalW as implemented in Bioedit (Hall, 1999).
(PDF)

S3 Fig Phylogenies of *MAT* genes. Distinct colors encompassing groups of strains indicate the *P*. *rhodozyma* populations to which the various strains belong. Population A: orange; Population B: red; Population C: green; Population D: blue. Nucleotide unrooted maximum likelihood phylogenies were inferred with General Time Reversible model and 1000 bootstrap replications on MEGA5.1 software. The trees with the highest log likelihood are shown with branch lengths measured in the number of substitutions per site. In the final datasets, *STE3-1*, *STE3-2*, *HD1* and *HD2* have 703, 735, 814 and 1441 positions respectively.
(PDF)

S4 Fig Bacterial two-hybrid assays. *E*. *coli* BHT101 transformants expressing various combinations of fusion proteins (C) growing on LB/ X-gal media plates (A).and on MacConkey/maltose medium plates (B).
(PDF)

S5 Fig Yeast two-hybrid assays. **A.** and **B.** Three independent diploid strains (X, Y and Z) expressing each of the combinations (D) of fusion proteins (C) growing on selective medium to assess adenine and histidine prototrophy (A) and indicator medium containing X-alpha GAL to assess expression of the *MEL1* reporter gene. Individual fusion proteins were unable to activate transcription of the reporter genes (E).
(PDF)

S6 Fig Plasmids used and constructed in this work. Plasmid pBS-HYG and pPR2TN were used to construct deletion fragments used in this work. Plasmids pJET1.2 and pUC18 were used to generate pJET1.2+ZEO and pUC18+rDNA+ZEO plasmids. Depicted in each plasmid map are the primers (indicated as MP followed by their designation number) and enzyme restriction sites used for the construction of the deletion fragments used in this work.
(PDF)

S7 Fig Southern blot analysis of selected deletion mutants. Autoradiographs of the results obtained for each mutant. Expected fragment sizes are shown.
(PDF)

S1 Table Amino acid identity (%) between different alleles of pheromone receptors from selected basidiomycete species. Proteins were aligned using ClustalW as implemented in Bioedit (Hall, 1999).
(PDF)

S2 Table Data from crosses of double and triple mutant strains. (PDF)

S3 Table Sporulation data. **A.** Sporulation data pertaining to plot E (Fig 1), plot C (Fig 3) and plot A (Fig 4). **B.** Sporulation data pertaining to plot G (Fig 1).
(PDF)

S4 Table List of plasmids and primers used in the Matchmaker Gold Yeast Two-Hybrid System. (PDF)

S5 Table List of putative homeodomain proteins retrieved from CBS 6938 genome. (XLSX)

S6 Table List of strains used in this work and relevant features. (PDF)

S7 Table Primers and plasmids used for the construction of all gene deletion fragments except for gene STE3-2. (PDF)

S8 Table Primers and plasmid used for the construction of the *STE3-2* deletion fragment by overlap extension PCR. (PDF)

S9 Table Primers and plasmid used for the construction of the Zeocin resistance cassette. (PDF)

S10 Table Primers and plasmid used for the construction of the complementation plasmids. (PDF)

S11 Table List of plasmids and primers used in the Bacterial Adenylate Cyclase Two-Hybrid System. (PDF)

S12 Table Accession numbers for all sequences obtained and used in this work. (PDF)

S1 File Sequence of synthetic Homeodomain genes. (PDF)
