# Supplementary figures and images for: Genetic Dissection of Sexual Reproduction in a Primary Homothallic Basidiomycete
Source: PLoS Genet. 2016 Jun 21;12(6):e1006110. doi: 10.1371/journal.pgen.1006110 (PMC4915694; doi:10.1371/journal.pgen.1006110)

## Sexual reproduction

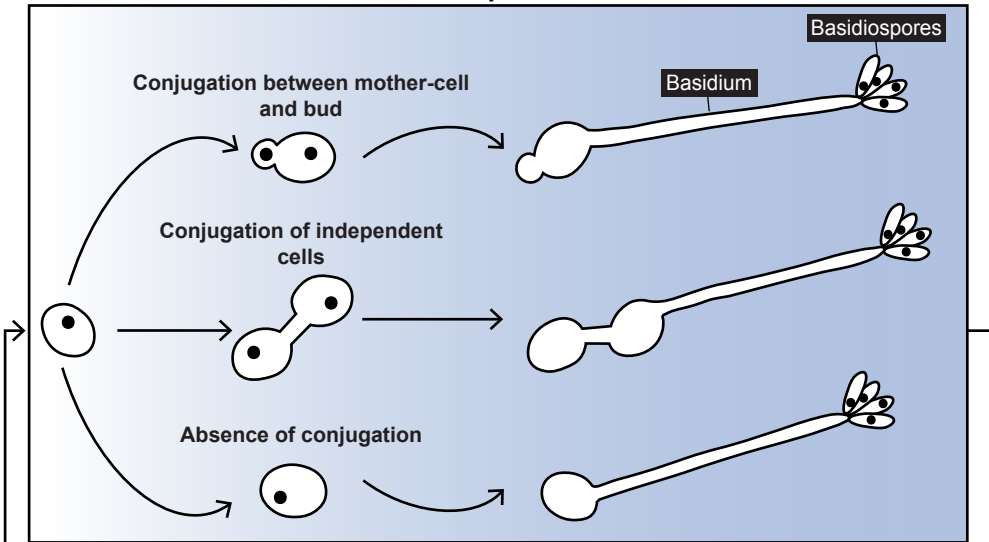

## Asexual reproduction

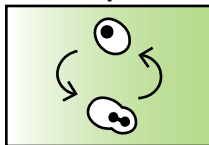

Supplement: S1 Fig — Vegetative cells propagate by budding. Nitrogen depletion and the presence of polyols trigger the formation of an aerial basidium that gives rise to apical basidiospores. Three distinct cellular events were observed to give rise to formation of the basidium: conjugation of independent cells, conjugation between mother-cell and bud (pedogamy) and single cells. (PDF) [file pgen.1006110.s001.pdf]

**STE3-1**

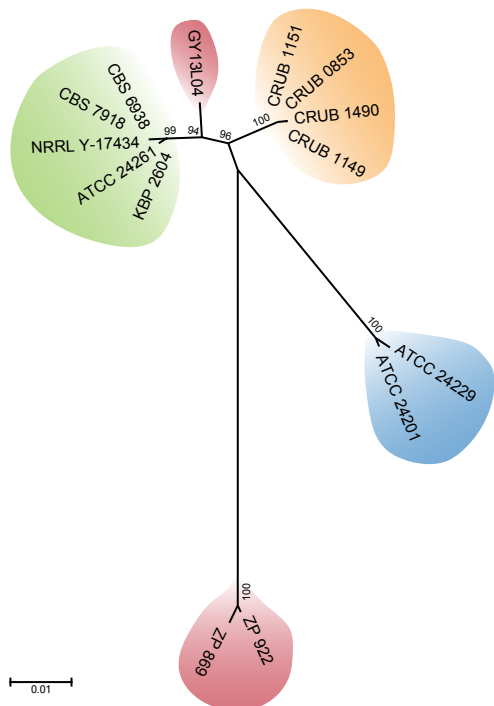

**STE3-2**

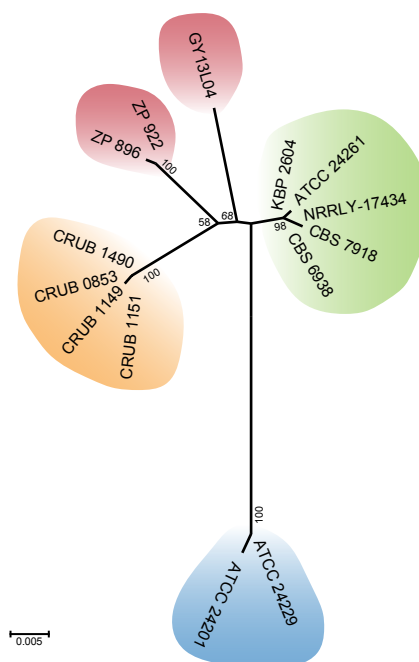

**HD1**

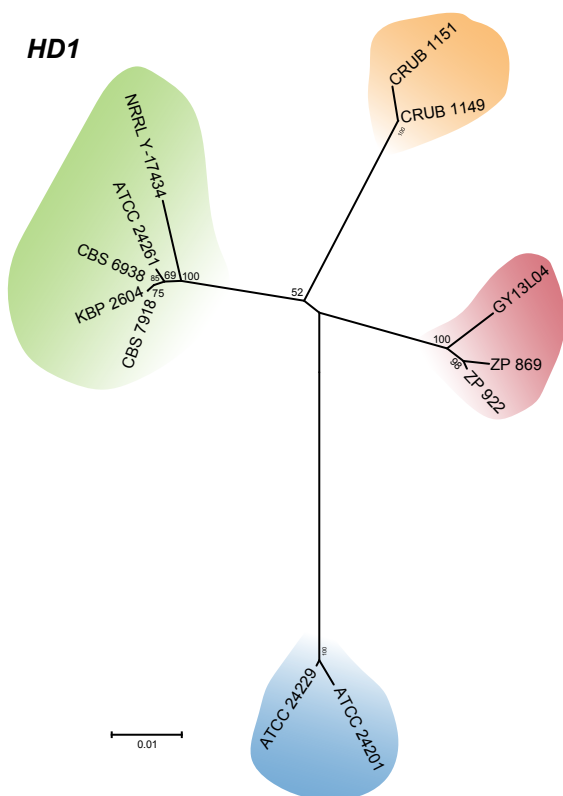

**HD2**

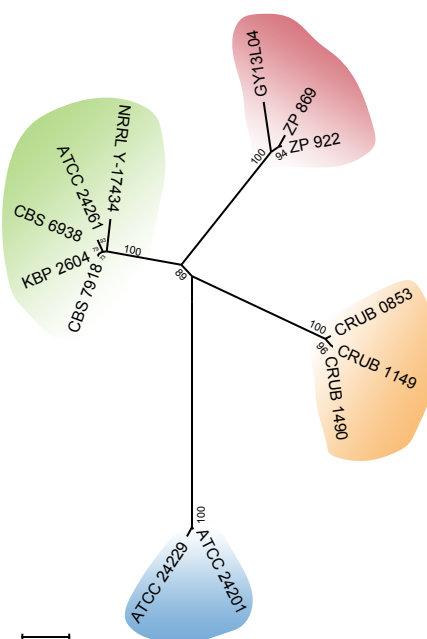

Supplement: S3 Fig — Distinct colors encompassing groups of strains indicate the P. rhodozyma populations to which the various strains belong. Population A: orange; Population B: red; Population C: green; Population D: blue. Nucleotide unrooted maximum likelihood phylogenies were inferred with General Time Reversible model and 1000 bootstrap replications on MEGA5.1 software. The trees with the highest log likelihood are shown with branch lengths measured in the number of substitutions per site. In the final datasets, STE3-1, STE3-2, HD1 and HD2 have 703, 735, 814 and 1441 positions respectively. (PDF) [file pgen.1006110.s003.pdf]

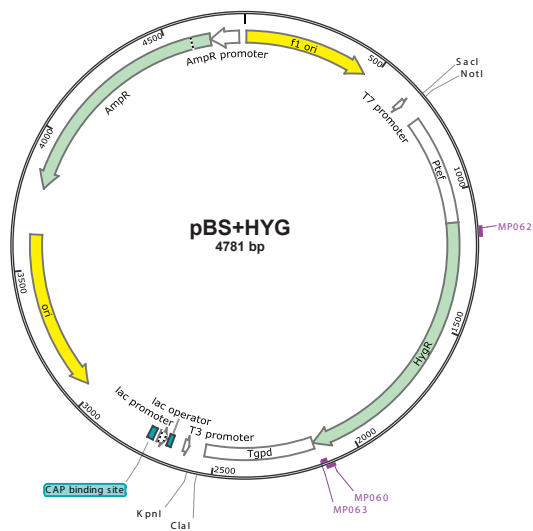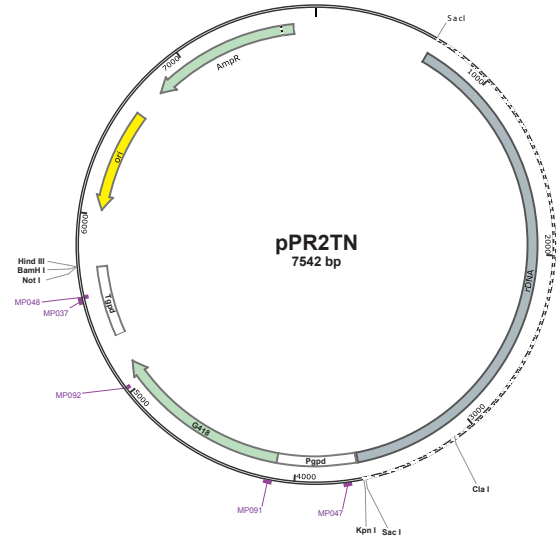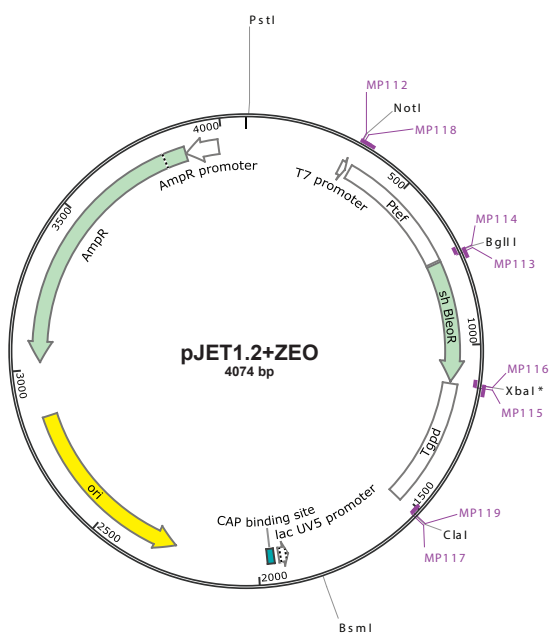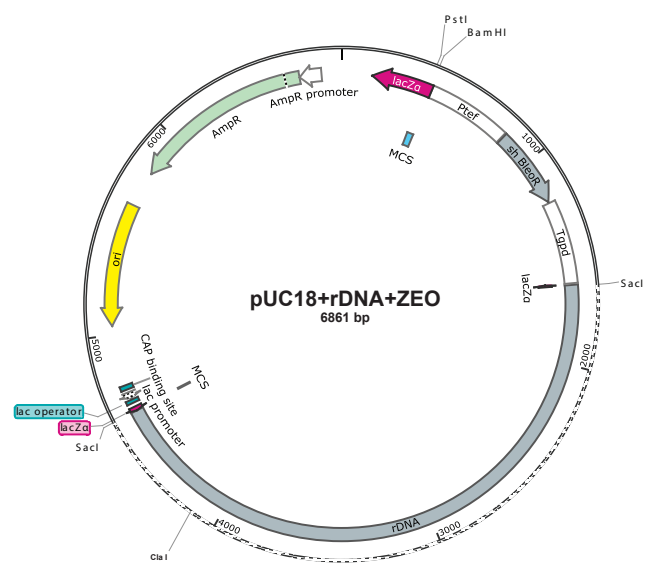

Supplement: S6 Fig — Plasmid pBS-HYG and pPR2TN were used to construct deletion fragments used in this work. Plasmids pJET1.2 and pUC18 were used to generate pJET1.2+ZEO and pUC18+rDNA+ZEO plasmids. Depicted in each plasmid map are the primers (indicated as MP followed by their designation number) and enzyme restriction sites used for the construction of the deletion fragments used in this work. (PDF) [file pgen.1006110.s006.pdf]
