# Supplementary material for: Genetic Dissection of Sexual Reproduction in a Primary Homothallic Basidiomycete
Source: PLoS Genet. 2016 Jun 21;12(6):e1006110. doi: 10.1371/journal.pgen.1006110 (PMC4915694; doi:10.1371/journal.pgen.1006110)
Supplement: S2 Fig — Sequences were retrieved from the available genomes of P. rhodozyma strains (CBS 7918, CBS 6938 and CRUB 1149) and obtained through this work (S12 Table). Alignment was performed using ClustalW as implemented in Bioedit (Hall, 1999). (PDF) [file pgen.1006110.s002.pdf]

S2_Fig.

```
                                        10        20        30        40        50        60        70        80        90       100
                              ....|....|....|....|....|....|....|....|....|....|....|....|....|....|....|....|....|....|....|....|
CBS6938_STE3-1_PROTEIN        MKDAIFPVFASIALLLLVLSCYPHFRTGNIGAIALVAWCFASNFVYLVDCLIYWDTVENLTPVWCDIMVKIQATTQTGLAAACLCINRRLAIISCSKQTS
CBS7918_STE3-1_PROTEIN        MKDAIFPVFASIALLLLVLSCYPHFRTGNIGAIALVAWCFASNFVYLVDCLIYWDTVENLTPVWCDIMVKIQATTQTGLAAACLCINRRLAIISCSKQTS
CRUB1149_STE3-1_PROTEIN       MKDAIFPVFASIALLLLVLSCYPHFRTGNIGAIALVAWCFASNFVYLVDCLFYWDTVENLTPVWCDIMVKIQATTQTGLAAACLCINRRLAIISRSKQTS
Clustal Consensus             ***************************************************:****************************************** *****

                                       110       120       130       140       150       160       170       180       190       200
                              ....|....|....|....|....|....|....|....|....|....|....|....|....|....|....|....|....|....|....|....|
CBS6938_STE3-1_PROTEIN        ATSKSRRWAFWSDIMICVLAPVIVAVVSYCVQSHRYNIVENFGCSGSPWMDVYAILGLHGSPVLLGAISFVYGAIAIYNFIAQRRRFQVVLQQNSSLNTS
CBS7918_STE3-1_PROTEIN        ATSKSRRWAFWSDIMICVLAPVIVAVVSYCVQSHRYNIVENFGCSGSPWMDVYAILGLHGSPVLLGAISFVYGAIAIYNFIAQRRRFQVVLQQNSSLNTS
CRUB1149_STE3-1_PROTEIN       ATSKSRRWAFWSDIMICVLAPVIVAVVSYCVQSHRYNIVENFGCSGSPWMDVYAILGLHGSPVLLGAISFVYGAIAIYNFIAQRRRFQVILQQNSSLNTS
Clustal Consensus             *****************************************************************************************:**********

                                       210       220       230       240       250       260       270       280       290       300
                              ....|....|....|....|....|....|....|....|....|....|....|....|....|....|....|....|....|....|....|....|
CBS6938_STE3-1_PROTEIN        RFVRLIGVAGVNIVISLLFAIRETVLTAHSVYPTVSWDYIHYDFDLVFTYDSFFLLGDPQAWVELNLSRWLPCVASFIYFAFFGMHEDMLSYYTYVWARL
CBS7918_STE3-1_PROTEIN        RFVRLIGVAGVNIVISLLFAIRETVLTAHSVYPTVSWDYIHYDFDLVFTYDSFFLLGDPQAWVELNLSRWLPCVASFIYFAFFGMHEDMLSYYTYVWARL
CRUB1149_STE3-1_PROTEIN       RFVRLIGVAGVNIVISLLFAIRETVLTAHSVYPTVSWDYIHYDFDLVFTYDSSFLLGDPQAWVELNLSRWLPCVASFIYFAFFGMHEDMLSYYTYVWARL
Clustal Consensus             **************************************************** ***********************************************

                                       310       320       330       340       350       360       370
                              ....|....|....|....|....|....|....|....|....|....|....|....|....|....|..
CBS6938_STE3-1_PROTEIN        SQALLRTKERIFGQPLTVHDPSQYPKLGTAVASPSECGWSQDKISDEEQVFPHSRTEISEKSNIDEGSLPRFEKICL
CBS7918_STE3-1_PROTEIN        SQALLRTKERIFGQPLTVHDPSQYPKLGTAVASPSECGWSQDKISDEEQVFPHSRTEISEKSNIDEGSLPRFEKICL
CRUB1149_STE3-1_PROTEIN       SQALLRTKERIFGQPLTVHDPSQYPKLGTAVASPSERGWSQDRISDEEQVFPHSRTEISEKSNTDEGSLPRFEKICL
Clustal Consensus             ************************************ *****:******************** *************
```

S2_Fig. (cont.)

```
                                   10        20        30        40        50        60        70        80        90       100
                         ....|....|....|....|....|....|....|....|....|....|....|....|....|....|....|....|....|....|....|....|
CBS6938_STE3-2_PROTEIN   MRDALFPIYSGLAILLLLMACGPHVRARNVGAISLMTWCMLANITYFINALVYQNTAEDLTPIWCDVVVKIQCAVQTGLAASCLCINRRLATISSPTQSR
CBS7918_STE3-2_PROTEIN   MRDALFPIYSGLAILLLLMACGPHVRARNVGAISLMTWCMLANITYFINALVYQNTAEDLTPIWCDVVVKIQCAVQTGLAASCLCINRRLATISSPTQSR
CRUB1149_STE3-2_PROTEIN  MRDALFPIYSGLAILLLLMACGPHVRARNVGAISLMTWCMLANITYFINALVYQNTAEDLTPIWCDVVVKIQCAVQTGLAASCLCINRRLATISSPTQSR
Clustal Consensus        ****************************************************************************************************

                                  110       120       130       140       150       160       170       180       190       200
                         ....|....|....|....|....|....|....|....|....|....|....|....|....|....|....|....|....|....|....|
CBS6938_STE3-2_PROTEIN   QTETSRQWAFWSDVAICLLPTTLVLIVSYCVQAHRYNIVENFGCFPATWLELYAILGLFVPPILCAAGSFICGGFAIYNFLAQRRRFQAVLQQHSSSLNS
CBS7918_STE3-2_PROTEIN   QTETSRQWAFWSDVAICLLPTTLVLIVSYCVQAHRYNIVENFGCFPATWLELYAILGLFVPPILCAAGSFICGGFAIYNFLAQRRRFQAVLQQHSSSLNS
CRUB1149_STE3-2_PROTEIN  QTETSRQWAFWSDVAICLLPTTLVLIVSYCVQAHRYNIVENFGCFPATWLELYAILGLFVPPILCAAGSFICGGFAIYNFLAQRRRFQAVLQQHSSSLNS
Clustal Consensus        ****************************************************************************************************

                                  210       220       230       240       250       260       270       280       290       300
                         ....|....|....|....|....|....|....|....|....|....|....|....|....|....|....|....|....|....|....|
CBS6938_STE3-2_PROTEIN   SRFLRLIGVAAVDMVLSLPFGIYEIIHNSYNLQPTYSWADLHHSFDLVQETDQSILNAQPGSWASINLSRWTTTLAAFIYFAFFGMHEDALSFHASTWNK
CBS7918_STE3-2_PROTEIN   SRFLRLIGVAAVDMVLSLPFGIYEIIHNSYNLQPTYSWADLHHSFDLVQETDQSILNAQPGSWASINLSRWTTTLAAFIYFAFFGMHEDALSFHASTWNK
CRUB1149_STE3-2_PROTEIN  SRFLRLIGVAAVDMVLSLPFGIYEIIHNSYNLQPTYSWVDLHHSFDIVQETDQSILNAQPGSWASINLSRWTTTLAAFIYFAFFGMHEDALSFHASTWNK
Clustal Consensus        **************************************.*******:*****************************************************

                                  310       320       330       340       350       360       370       380
                         ....|....|....|....|....|....|....|....|....|....|....|....|....|....|....|....|....|
CBS6938_STE3-2_PROTEIN   ITAAFSYIWMRAFGTSEPPSYDSSHLTELGTTVASVSDLNRFCKSSKDEEAYFAYENMIGSDKSDSDKGVDSKISVTVRIQKTIS
CBS7918_STE3-2_PROTEIN   ITAAFSYIWMRAFGTSEPPSYDSSHLTELGTTVASVSDLNRFCKSSKDEEAYFAYENMIGSDKSDSDKGVDSKISVTVRIQKTIS
CRUB1149_STE3-2_PROTEIN  ITAAFSYIWMRAFGTSEPPSYDSSHLTELGTTVASVSDLNRFCKSSKDEEAYFAYENMIGSDKSDSDIGVDSKISVTVRIQKTIS
Clustal Consensus        ******************************************************************* *****************
```

S2_Fig. (cont.)

```
                                10        20        30        40        50        60        70        80        90       100
                        ....|....|....|....|....|....|....|....|....|....|....|....|....|....|....|....|....|....|....|....|
CBS6938_STE3-1_PROTEIN  MKDAIFPVFASIALLLLVLSCYPHFRTGNIGAIALVAWCFASNFVYLVDCLIYWDTVENLTPVWCDIMVKIQATTQTGLAAACLCINRRLAIISCSKQTS
CB6938_STE3-2_PROTEIN   MRDALFPIYSGLAILLLLMACGPHVRARNVGAISLMTWCMLANITYFINALVYQNTAEDLTPIWCDVVVKIQCAVQTGLAASCLCINRRLATISSPTQSR
Clustal Consensus       *:**:**:::.:*:***:::* **.*: *:***:*::**: :*:.*:::.*:* :*.*:***:***::****.:.******:********* **...*:

                               110       120       130       140       150       160       170       180       190       200
                        ....|....|....|....|....|....|....|....|....|....|....|....|....|....|....|....|....|....|....|....|
CBS6938_STE3-1_PROTEIN  ATSKSRRWAFWSDIMICVLAPVIVAVVSYCVQSHRYNIVENFGCSGSPWMDVYAILGLHGSPVLLGAISFVYGAIAIYNFIAQRRRFQVVLQQNSS-LNT
CB6938_STE3-2_PROTEIN   QTETSRQWAFWSDVAICLLPTTLVLIVSYCVQAHRYNIVENFGCFPATWLELYAILGLFVPPILCAAGSFICGGFAIYNFLAQRRRFQAVLQQHSSSLNS
Clustal Consensus        *..**:******: **:*...:* :******:***********  :.*:::******. .*:* .* **: *.:*****:*******.****:** **:

                               210       220       230       240       250       260       270       280       290       300
                        ....|....|....|....|....|....|....|....|....|....|....|....|....|....|....|....|....|....|....|....|
CBS6938_STE3-1_PROTEIN  SRFVRLIGVAGVNIVISLLFAIRETVLTAHSVYPTVSWDYIHYDFDLVFTYDSFFLLGDPQAWVELNLSRWLPCVASFIYFAFFGMHEDMLSYYTYVWAR
CB6938_STE3-2_PROTEIN   SRFLRLIGVAAVDMVLSLPFGIYEIIHNSYNLQPTYSWADLHHSFDLVQETDQSILNAQPGSWASINLSRWTTTLAAFIYFAFFGMHEDALSFHASTWNK
Clustal Consensus       ***:******.*::*:** *.* * : .::.: ** **  :*:.****   *. :* .:* :*..:***** . :*:************ **::: .* :

                               310       320       330       340       350       360       370       380
                        ....|....|....|....|....|....|....|....|....|....|....|....|....|....|....|....|....|
CBS6938_STE3-1_PROTEIN  LSQALLRTKERIFGQP-LTVHDPSQYPKLGTAVASPSECGWSQDKISDEEQVFPHSRTEISEKSNIDEGS------LPRFEKICL
CB6938_STE3-2_PROTEIN   ITAAFSYIWMRAFGTSEPPSYDSSHLTELGTTVASVSDLNRFCKSSKDEEAYFAYENMIGSDKSDSDKGVDSKISVTVRIQKTIS
Clustal Consensus       :: *:     * ** .  . :*.*: .:***:*** *: .   .. .***  *.:..   *:**: *:*         *::*
```

S2_Fig. (cont.)

```
                                      10        20        30        40
                              ....|....|....|....|....|....|....|....|...
CBS6938_MFA1_PROTEIN          MDAFTAIFTPFVASTNESPAASSAKGETKEWTPGMPTSTCVIA
CBS7918_MFA1_PROTEIN          MDAFTAIFTPFVASTNESPAASSAKGETKEWTPGMPTSTCVIA
CRUB1149_MFA1_PROTEIN         MDAFTAIFTPFVASTNESPAASSAKGETKEWTPGMPTSTCVIA
Clustal Consensus             *******************************************
```

S2_Fig. (cont.)

```
                                10        20        30        40
                        ....|....|....|....|....|....|....|....|.
CBS7918_MFA2_PROTEIN    MDVFTTIFTPFVASTETELTSEAVGERPESKQMFTNFCVIA
CRUB1149_MFA2_PROTEIN   MDVFTTIFTPFVASTETELTSEAVGERPESKQMFTNFCVIA
CBS6938_MFA2_PROTEIN    MDVFTTIFTPFVASTETELTSEAVGERPESKQMFTNFCVIA
Clustal Consensus       *****************************************
```

S2_Fig. (cont.)

```
                                10        20        30        40        50        60        70        80        90       100
                        ....|....|....|....|....|....|....|....|....|....|....|....|....|....|....|....|....|....|....|....|
CBS6938_HD1_protein     MEPSPASDLSARLDSLVETFLISIRAGVPIELSQIYDAFAEERCKARSQGLLTFAFQEEISASVGFLSTLASCMMNIPEGNKDFQTTVIGILNNANNTPE
CBS7918_HD1_protein     MEPSPASDLSARLDSLVETFLISIRAGVPIELSQIYDAFAEERCKARSQGLLTFAFQEEISASVGFLSTLASCMMNIPEGNKDFQTTVIGILNNANNTPE
CRUB1149_HD1_protein    MEPSPASDLSARLDSLVETFLNSIRAGVPIELSQIYDAFAEERHKARSEGLLTFAFQEEISASVGFLSTLTSCMMNIPEGNKDFQTTVIGILNNANNTPE
CRUB 1151 HD1_protein   MEPSPASDLSARLDSLVETFLNSIRAGVPIELSQIYDAFAEERHKARSEGLLTFAFQEEISASVGFLSTLNSCMMNIPEGNKDFQTTVIGILNNANNTPE
GY13L04 HD1_protein     MESSPASDLSARLDSLVETFLNSIRAGVPIELSQIYDAFAEERHKARSEGLLTFAFQEEISASVGFLSTLASCMMNIPEGNKDFQTTVIGILNNANNTPE
ZP 869 HD1_protein      MEPSPASDLSARLDSLVETFLNSIRAGVPIELSQIYDAFAEERHKARSEGLLTFAFQEEISASVGFLSTLASCMMNIPEGNKDFQTTVIGILNNANNTPE
ZP 922 HD1_protein      MEPSPASDLSARLDSLVETFLNSIRAGVPIELSQIYDAFAEERHKARSEGLLTFAFQEEISASVGFLSTLASCMMNIPEGNKDFQTTVIGILNNANNTPE
ATCC 24229 HD1_protein  MEPSPASDLSARLDSLVETFLNSIRAGVPIELSQIYDAFAEERRKARSEGLLTFAFQEEISASVGFLSTLASCMMNIPEGNKDFQTTVIGILNDANNTPE
ATCC24201_HD1_protein   MEPSPASDLSARLDSLVETFLNSIRAGVPIELSQIYDAFAEERRKARSEGLLTFAFQEEISASVGFLSTLASCMMNIPEGNKDFQTTVIGILNDANNTPE
Clustal Consensus       **.****************** ********************* ****:********************* **********************:******

                               110       120       130       140       150       160       170       180       190       200
                        ....|....|....|....|....|....|....|....|....|....|....|....|....|....|....|....|....|....|....|....|
CBS6938_HD1_protein     LCPSRNLVSPPKILASKADSGRSMRPRTEHPSSFRHLRDWFIVHLDHPYPSAQEKEELALARNLTKNSINLWFNNMRRRSGWMDFVRTHALNNQTRMKEL
CBS7918_HD1_protein     LCPSRNLVSPPKILASKADSGRSMRPRTEHPSSFRHLRDWFIVHLDHPYPSAQEKEELALARNLTKNSINLWFNNMRRRSGWMDFVRTHALNNQTRMKEL
CRUB1149_HD1_protein    LCPSRNLVSPPKGLASKADSGRSMRPRTEHPSSFRHLRDWFIVHLDHPYPSAQEKEELALARNLTKNSINLWFNNMRRRSGWMDFVRTHALNNQTRMKEL
CRUB 1151 HD1_protein   LCPSRNLVSPLKGLASKADSGRSMRPRTEHPSSFRHLRDWFIVHLDHPYPSAQEKEELALARNLTKNSINLWFNNMRRRSGWMDFVRTHALNNQTRMKEL
GY13L04 HD1_protein     LCPSRNLISPPKILASKADSGRSMRPRTEHPSSFRHLRDWFIVHLDHPYPSAQEKEELALARNLTKNSINLWFNNMRRRSGWMDFVRTHALNNQTRMKEL
ZP 869 HD1_protein      LCPSRNLISPPKILASKADSGRSMRPRTEHPSSFRHLRDWFIVHLDHPYPSAQEKEELALARNLTKNSINLWFNNMRRRSGWMDFVRTHALNNQTRMKEL
ZP 922 HD1_protein      LCPSRNLISPPKILASKADSGRSMRPRTEHPSSFRHLRDWFIVHLDHPYPSAQEKEELALARNLTKNSINLWFNNMRRRSGWMDFVRTHALNNQTRMKEL
ATCC 24229 HD1_protein  LCPSRNLISPPKILASKADSGRSMRPRTEHPSSFRHLRDWFIVHLDHPYPSAQEKEELALAKNLTKNSINLWFNNMRRRSGWMDFMRTHAHNNQTRMKEL
ATCC24201_HD1_protein   LCPFRNLISPPKILASKADSGRSMRPRTEHPSSFRHLRDWFIVHLDHPYPSAQEKEELALAKNLTKNSINLWFNNMRRRSGWMDFMRTHAHNNQTRMKEL
Clustal Consensus       *** ***:** * ************************************************:***********************:**** *********

                               210       220       230       240       250       260       270       280       290       300
                        ....|....|....|....|....|....|....|....|....|....|....|....|....|....|....|....|....|....|....|....|
CBS6938_HD1_protein     VLLHRSLPAVERSHPDLPFELRQQLDRIRDHVDEKGHDRVRDWMTGALDYKDGFGVPAEGIGRSPEKKNEVNKDTAKSGSSSILYKQAFTAKDAQPWLTS
CBS7918_HD1_protein     VLLHRSLPAVERSHPDLPFELRQQLDRIRDHVDEKGHDRVRDWMTGALDYKDGFGVPAEGIGRSPEKKNEVNKDTAKSGSSSILYKQAFTAKDAQPWLTS
CRUB1149_HD1_protein    VLLHRSLPAVEQCHPDLPFELRQQLDRIRDHVDEKGHDRVRDWMTGALDYRDGFSISAEGIGRSPEKKNEVNKDTAKPGSSSNLYKQAFAAKDAQPWLTS
CRUB 1151 HD1_protein   VLLHRSLPAVEQSHPDLPFELRQQLDRIRDHVDEKGHDRVRDWMTGALDYRDGFSISAEGIGRSPEKKNEVNKDTAKPGSSSNLYKQAFAAKDAQPWLTS
GY13L04 HD1_protein     VLLHRSLPAVERSHPDLPFELRQLLDRIQDHVDEKGHDRVRDWMTGALDYRDGFSVPAEGIGRSPEKKNEVNKDTVKFGPSSNLYKQAFTAKDAQPWHTS
ZP 869 HD1_protein      VLLHRSLPAVERSHPDLPFELRQQLDRIQDHVDEKGHDRVRDWMTGALDYRDGFSVPAEGIGRSPEKKNEVNKDTVKFGPSSNLYKQAFTAKDAQPWHTS
ZP 922 HD1_protein      VLLHRSLPAVERSHPDLPFELRQQLDRIQDHVDEKGHDRARDWMKGALDYRDGFSVPAEGIGRSPEKKNEVNKDTVKFGPSSNLYKQAFTAKDAQPWHTS
ATCC 24229 HD1_protein  VLLHRSLPAVERSHPDLPFELRQQLDRIRDHVDEKGHDRVRDWMTGALEYREGFSIPAEGIGRSPEKKNEVNKATTKSGSSSDLYKQAFTAKDAQPWLTS
ATCC24201_HD1_protein   ILLHRSLPAVERSHPDLPFELRQQLDRIRDHVDEKGHDRVRDWMTGALEYREGFSIPAEGIGRSPEKKNEVNKATTKSGSSSDLYKQAFTAKDAQPWLTS
Clustal Consensus       :**********:.********** ****:**********.****.***:*::**.:.**************** *.* *.** ******:******* **
```

```
                                   310       320       330       340       350       360       370       380       390       400
                              ....|....|....|....|....|....|....|....|....|....|....|....|....|....|....|....|....|....|....|....|
CBS6938_HD1_protein           YPTAASGLIGYPSSIPLANCPTPKSSKRLFSDCGPHLAAPIDPVNKYHQLINLSSGPTQDLEGSLLTHQPLSAYAYYPTHPQSQNPLPMLGPTPYRSSSD
CBS7918_HD1_protein           YPTAASGLIGYPSSIPLANCPTPKSSKRLFSDCGPHLAAPIDPVNKYHQLINLSSGPTQDLEGSLLTHQPLSAYAYYPTHPQSQNPLPMLGPTPYRSSSD
CRUB1149_HD1_protein          YPTAASGLIGYPSSVPLANCSNPKSSKRSLSDCGPHLAAPIDPVNKYHQLMNVSSGPNQDLEGSLLTHQPLPTYAYYPTHPQSQNSLPTLGPTPYHPSSD
CRUB 1151 HD1_protein         YPTAASGLIGYPSSVPLANCSNPKSSKRSFSDCGPHLAAPIDPVNKYHQLMNVSSGPNQDLEGSLLTHQPLSTYAYYPIYPQSQNSLPTLGPTPYHPSSD
GY13L04 HD1_protein           YPTAASGLIGYPSSIPLANGPNTKSSKRSFSDCGPHLAASIDPVNKYHQLMNLSSGPIQDLEGSLLTHQPLSAYAYYPTHPQSQNPLPMLGPTPYHSSSD
ZP 869 HD1_protein            YPTSASGLIGYPSSIPLANGPNTKSSKRSFSDCGPHPAAPIDPVNKYHQLMNLSSGPIQDLEGSLLTHQPLSAYAYYPTHPQSQNPLPMLGPTPYHSSSD
ZP 922 HD1_protein            YPTAASGLIGYPSSIPLANGPNTKSSKRSFSDCGPHPAAPIDPVNKYHQLMNLSSGPIQDLEGSLLTHQPLSAYAYYPTHPQSQNPLPMLGPTPYHSSSD
ATCC 24229 HD1_protein        YPTAASGLIGYQSPIPLANCPNPKSSKRSFSDCGPHLAAPVDPVNKYHQLMNLSSRPIQDLEGSLFTHQPLSAYAYYPTHPQSQNPLPMLSPTPYHSSSD
ATCC24201_HD1_protein         YPTAASGLIGYQSPIPLANCPNPKSSKRSFSDCGPHLAAPVDPVNKYHQLMNLSSRPIQDLEGSLFTHQPLSAYAYYPTHPQSQNPLPMLSPTPYHSSSD
Clustal Consensus             ***:******* *.:**** ...***** :****** **.:*********:*:** * *******:*****.:***** :*****.** *.****:.***

                                   410       420       430       440       450       460       470       480       490       500
                              ....|....|....|....|....|....|....|....|....|....|....|....|....|....|....|....|....|....|....|....|
CBS6938_HD1_protein           SQYPAYDLKAPIRAQTWLPSDPAIDPSNGPNIHQRSVSPLPSRAKSTVSSLATSTTLQSTRFSVNESAGNELAAKLEALRIKKEALKKEKEQLRAEAEAL
CBS7918_HD1_protein           SQYPAYDLKAPIRAQTWLPSDPAIDPSNGPNIHQRSVSPLPSRAKSTVSSLATSTTLQSTRFSVNESAGNELAAKLEALRIKKEALKKEKEQLRAEAEAL
CRUB1149_HD1_protein          SQYPAYDLKAPIRAQTWLPSDPAIDPSNGPNIHQRSVSPLPSRAKSTVSSLATSTTSQSTRSSANESAGNELAAKLEALRIKKEALKKEKEQLRAEAEAL
CRUB 1151 HD1_protein         SQYPAYDLKAPIRAQTWLPSDPAIDPSNGPNIHQRSVSPLPSRAKSTVSSLATSTTSQSTRSSANESAGNELAAKLEALRIKKEALKKENEQLRAEAEAL
GY13L04 HD1_protein           CQYPAYDLKAPIRAQTWLPSDPAIDPTNGPNIHQRSVSPLPSRAKSTASSLATSSTLQSTRPSANESAGNELAAKLEALRIKKEALKKEKEQLRAEAEAL
ZP 869 HD1_protein            CQYPAYDLKAPIRPQTWLPSDPAIDPTNGPNIHQRSVSPLPSRAKSTVSSLATSSTLQSTRPSANESADNELAAKLEALRIKKEALKKEKEQLRAEAEAL
ZP 922 HD1_protein            CQYPAYDLKAPIRAQTWLPSDPAIDPTNGPNIHQRSVSPLPSRAKSTVSSLATSSTLQSTRPSANESAGNELAAKLEALRIKKEALKKEKEQLRAEAEAL
ATCC 24229 HD1_protein        PQYPAYDLKAPIRAQTWLPSDPAIDLSNGPNIHQRSVSPLPSRAKSTVSSLATSTTLQSTRSAANESAGNELAAKLEALRIKKEALKKEKEQLRAEAEAL
ATCC24201_HD1_protein         PQYPAYDLKAPIRAQTWLPSDPAIDLSNGPNIHQRSVSPLPSRAKSTVSSLATSTTLQSTRSAANESAGNELAAKLEALRIKKEALKKEKEQLRAEAEAL
Clustal Consensus              ************.*********** :********************.******:* **** :.****.********************:**********

                              ....|.
CBS6938_HD1_protein           GEVFDC
CBS7918_HD1_protein           GEVFDC
CRUB1149_HD1_protein          GEVFDC
CRUB 1151 HD1_protein         GEVFDC
GY13L04 HD1_protein           GEVFDC
ZP 869 HD1_protein            GEVFDC
ZP 922 HD1_protein            GEVFDC
ATCC 24229 HD1_protein        GEVFDC
ATCC24201_HD1_protein         GEVFDC
Clustal Consensus             ******
```

Homeodomain determined using Pfam server

S2_Fig. (cont.)

```
                            10        20        30        40        50        60        70        80        90       100
                    ....|....|....|....|....|....|....|....|....|....|....|....|....|....|....|....|....|....|....|....|
CRUB1490_HD2_protein    MYSFTKLPQKHTGLGDVFDSNLDSLSSKEFALEDLYLPIPTSICETLEELGTPHTLARNLGALYVSRAMALRELSLSQFDTAFPAERTFLLKANSLKLKV
CRUB1149_HD2_protein    MYSFTKLPQKHTGLGDVFDSNLDSLSSKEFALEDLYLPIPTSICETLEELGTPHTLARNLGALYVSRAMALRELSLSQFDTAFPAERTFLLKANSLKLKV
CRUB0853_HD2_protein    MYSFTKLPQKYTGLGDVFDSNLDSLSSKKFALEDLYLPIPTSICETLEELGTPHTLARNLGALYVSRAMALRELSLSQFDTAFPAERTFLLEANSLKLKV
GY13L04_HD2_protein     MYNFTKLPQKCTGLGDVFDFNLDSLSSKEFALEDLYLPIPTSICETLEELGTPHTLARNLGALYLSRATALRELSLSQFDTAFPAERTFLLEANSLKLRV
ZP869_HD2_protein       MYNFTKLPQKYTGLGDVFDSNLDSLSSKEFALEDLYLPIPTSICETLEELGTPHTLARNLGALYLSRATTLRELSLSQFDTAFPAERTFLLEANSLKLRV
ZP922_HD2_protein       MYNFTKLPQKYTGLGDVFDSNLDSLSSKEFALEDLYLPIPTSICETLEELGTPHTLARNLGALYLSRATTLRELSLSQFDTAFPAERTFLLEANSLKLRV
CBS7918_HD2_protein     MYSFTKLPQKYTGLGDVFDSNIDSLSSKEFALEDLYLPIPTSICETLEELGTPHTLARNLGALYLSRAMALRELSLSQFDTAFPAERTFLLEANSLKLKV
CBS6938_HD2_protein     MYSFTKLPQKYTGLGDVFDSNIDSLSSKEFALEDLYLPIPTSICETLEELGTPHTLARNLGALYLSRAMALRELSLSQFDTAFPAERTFLLEANSLKLKV
ATCC24261_HD2_protein   MYSFTKLPQKYTGLGDVFDSNIDSLSSKEFALEDLYLPIPTSICETLEELGTPHTLARNLGALYLSRAMALRELSLSQLDTAFPAERTFLLEANSLKLKV
NRRLY17434_HD2_protein  MYSFTKLPQKYTGLGDVFDSNIDSLSFKEFALEDLYLPIPTSICETLEELGTPHTLARNLGALYLSRAMALRELSLSQFDAAFPAERTFLLEANSLKLKV
KBP2604_HD2_protein     MYSFTKLPQKYTGLGDVFDSNIDSLSSKEFALEDLYLPIPTSICETLEELGTPHTLARNLGALYLSRAMALRELSLSQFDTAFPAERTFLLEANSLKLKV
ATCC24201_HD2_protein   MYSFTKLPQKYTGLGDVFDSNRDPLSSKEFALEDLYLPIPPSICETLEELGTPHTLARSLGALYLSRAIALRDLSLSQFDAAFPAERTFLLEANSLKLKV
ATCC24229_HD2_protein   MYSFTKLPQKYTGLGDVFDSNRDPLSSKEFALEDLYLPIPPSICETLEELGTPHTLARSLGALYISRAIALRDLSLSQFDAAFPAERTFLLEANSLKLKV
Clustal Consensus       **.******* ******** * *.** *:***********.****************.*****:*** :**:*****:*:**********:******:*

                           110       120       130       140       150       160       170       180       190       200
                    ....|....|....|....|....|....|....|....|....|....|....|....|....|....|....|....|....|....|....|
CRUB1490_HD2_protein    FKGLLKATLEHRYSMELFHHQETIIQLAGVWTSDDEQAPVSLRRSSFTKNTIDTLQALYDLNQYPNPIELKTIAVKVGLQPKQIRAWFQNRRNRRSSPAR
CRUB1149_HD2_protein    FKGLLKATLEHRYSMELFHHQETIIQLAGVWTSDDEQAPVSLRRSSFTKNTIDTLQALYDLNQYPNPIELKTIAVKVGLQPKQIRAWFQNRRNRRSSPAR
CRUB0853_HD2_protein    FKGLLKATLEHRYSMELFHHQETIIQLAGVWTSDDEQAPVSLRRSSFTKNTIDTLQALYDLNQYPNPIELKTIAVKVGLQPKQIRAWFQNRRNRRSSPAR
GY13L04_HD2_protein     FKGLLKATLEHRYSMELFHHQETIIQLAGIWTSDDEQAPVSVRRSSFTKNTIDTLQALYDLNQYPNPIELKTIAVKVGLQPKQIRAWFQNRRNRRSSPAR
ZP869_HD2_protein       FKGLLKATLEHRYSMELFHHQETIIQLAGIWTSDDEQAPVSVRRSSFTKNTIDTLQALYDLNQYPNPIELKTIAVKVGLQPKQIRAWFQNRRNRRSSPAR
ZP922_HD2_protein       FKGLLKATLEHRYSMELFHHQETIIQLAGIWTSDDEQAPVSVRRSSFTKNTIDTLQALYDLNQYPNPIELKTIAVKVGLQPKQIRAWFQNRRNRRSSPAR
CBS7918_HD2_protein     FKGLLKATLENRYSMELFHHHETIIQLAGVWTSDDEQAPVSVRRSSFTKNTIDTLQALYDLNQYPNPIELKTIAVKVGLQPKQIRAWFQNRRNRRSSPAR
CBS6938_HD2_protein     FKGLLKATLENRYSMELFHHHETIIQLAGVWTSDDEQAPVSVRRSSFTKNTIDTLQALYDLNQYPNPIELKTIAVKVGLQPKQIRAWFQNRRNRRSSPAR
ATCC24261_HD2_protein   FKGLLKATLENRYSMELFHHHETIIQLAGVWTSDDEQAPVSVRRSSFTKNTIDTLQALYDLNQYPNPIELKTIAVKVGLQPKQIRAWFQNRRNRRSSPAR
NRRLY17434_HD2_protein  FKGLLKATLENRYSMELFHHQETIIQLAGVWTSDDEQAPVSVRRSSFTKNTIDTLQALYDLNQYPNPIELKTIAVKVGLQPKQIRAWFQNRRNRRSSPAR
KBP2604_HD2_protein     FKGLLKATLENRYSMELFHHHETIIQLAGVWTSDDEQAPVSVRRSSFTKNTIDTLQALYDLNQYPNPIELKTIAVKVGLQPKQIRAWFQNRRNRRSSPAR
ATCC24201_HD2_protein   FKGLLKATLEHRYSMELSHHQETIIQLAGVWTSDDEQAPVSVRRSSFTKNTIDTLQALYDLNQYPNPIELKTIAVKVGLQPKQIRAWFQNRRNRRSSPAR
ATCC24229_HD2_protein   FKGLLKATLEHRYSMELSHHQETIIQLAGVWTSDDEQAPVSVRRSSFTKNTIDTLQALYDLNQYPNPIELKTIAVKVGLQPKQIRAWFQNRRNRRSSPAR
Clustal Consensus       **********:****** **:********:**********:***********************************************************
```

```
                                       210       220       230       240       250       260       270       280       290       300
                               ....|....|....|....|....|....|....|....|....|....|....|....|....|....|....|....|....|....|....|....|
CRUB1490_HD2_protein           SHKKPLKISTRATGASDLKFNSVRKRCSRRTASIEKERKTEGVSSQLGSPIFLRTNKTPYVPQSEGRSRVGNSLVAGLFDTSPNEQDAYALWSAVVSDSS
CRUB1149_HD2_protein           SHKKPLKISTRATGASDLKFNSVRKRCSRRTASIEKERKTEGVSSQLGSPIFLRTNKTPYVPQSEGRSRVGNSLVAGLFDTSPNEQDAYALWSAVVSDSS
CRUB0853_HD2_protein           SHKKPLKISTRATGASDLKFNSVRKRCSRRTASIEKERKTEGVSSQLGSPIFLRTNKTPYVPQSEGRSRVGNSLVAGLFDTSPNEQDAYALWSAVVSDSS
GY13L04_HD2_protein            SHKKFLKISTRAAVASDLKFNSARKRCSRRTTSIEKERKTESVPCQLGSPIFLQTKKTPYVQQSEGRSRVGNSLVAGLFDTSSNEQDAYALWSAVVSDHS
ZP869_HD2_protein              SHKKFLKISTRAAVASDLKFNSARKRCSRRTTSIEKERKTESVPCQLGSPIFLQTKKTPYVPQSEGGSRVGNSLVAGLFDTSSNEQDAYALWSAVVSDHS
ZP922_HD2_protein              SHKKFLKISTRAAVASDLKFNSARKRCSRRTTSIEKERKTESVPCQLGSPIFLQTKKTPYVPQSEGGSRVGNSLVAGLFDTSSNEQDAYALWSAVVSDHS
CBS7918_HD2_protein            SHKKPLKISTRATVASDLKFNSVRKRCSRRTTSIEKERKTEGVPSQLGSPIFLRTNKTPYVPQSEGRSRVGNSLVAGLFDTSPNEQDAYALWSAVVSDRS
CBS6938_HD2_protein            SHKKPLKISTRATVASDLKFNSVRKRCSRRTTSIEKERKTEGVPSQLGSPIFLQTNKTPYVPQSEGRSRVGNSLVAGLFDTSPNEQDAYALWSAVVSDRS
ATCC24261_HD2_protein          SHKKPLKISTRATVASDLKFNSVRKRCSRRTTSIEKERKTEGVPSQLGSPIFLQTNKTPYVPQSEGRSRVGNSLVAGLFDTSPNEQDAYALWSAVVSDRS
NRRLY17434_HD2_protein         SHKKPLKISTRATVASDLKFNSARKRCSRRTTSIEKERKTEGVPSQLGSPIFLQTNKTPYVPQSEGRSRVGNSLVAGLFDTSPNEQDAYALWSAVVSDRS
KBP2604_HD2_protein            SHKKPLKISTRATVASDLKFNSVRKRCSRRTTSIEKERKTEGVPSQLGSPIFLRTNKTPYVPQSEGRSRVGNSLVAGLFDTSPNEQDAYALWSAVVSDRS
ATCC24201_HD2_protein          SHKKPLKISTRATFASDPKFNSVRKRCFRRTSSIEKERKTEGVPSQLGSPIFLQTNKTPYVPQSEGRSRVGHSLVAGLFDTSPNEQDAYALWSAVLSDRS
ATCC24229_HD2_protein          SHKKPLKISTRATFASDPKFNSVRKRCFRRTSSIEKERKTEGVPSQLGSPIFLQTNKTPYVPQSEGRSRVGHSLVAGLFDTSPNEQDAYALWSAVISDRS
Clustal Consensus              **** *******: *** ****.**** ***:*********.*..********:*:***** **** ****:**********.************:** *

                                       310       320       330       340       350       360       370       380       390       400
                               ....|....|....|....|....|....|....|....|....|....|....|....|....|....|....|....|....|....|....|....|
CRUB1490_HD2_protein           SAYANSKQVPQIESSLAFAIPEPVNSMSAQRSKNHFNGCSKSSSFHPVFPPVFNPSETMGVDPVDWTMVFAASDSRNIAPRSEKPSARWETKTGLEVEDA
CRUB1149_HD2_protein           SAYANSKQVPQIESSLAFAIPEPVNSMSAQRSKNHFNGCSKSSSFHPVFPPVFNPSETMGVDPVDWTMVFAASDSRNIAPRSEKPSARWETKTGLEVEDA
CRUB0853_HD2_protein           SAYANSKQVPQIESSLAFAIPEPVNSMSAQRSKNHFNGCSKSSSFHPVFPPVFNPSETMGVDPVDWTMVFAASDSRNIAPRSEKPSARWETKTGLEVEDA
GY13L04_HD2_protein            SAYANSKHVPQIESSLAFAIPEPADSMSAQRSENHLNGCSKASSSHSAFPPVFSSSETMEVDPVDWTMVFAASDSRNIAPRSEKPSARWETKKGLEVEDA
ZP869_HD2_protein              SAYANSKHVPQIESSLAFAIPEPADSMSAQRSENHLNGCSKSSSSHPAFPPVFSSSETMEVDPVDWTMVFAASDSRNIAPRSEKPSARWETKKGLEVEDA
ZP922_HD2_protein              SAYANSKHVPQIESSLAFAIPEPADSMSAQRSENHLNGCSKSSSSHPAFPPVFSSSETMEVDPVDWTMVFAASDSRNIAPRSEKPSARWETKKGLEVEDA
CBS7918_HD2_protein            SAYANSKHVPQIEPSLAFAIPEPADSMSAQRSENHFNSCSKSSSSHPAFPPVFGSSETMEVDPVDWTMVFAASDSRNIAPRSEKPSARWETKTGLEVEDA
CBS6938_HD2_protein            SAYANSKHVPQIEPSLAFAIPEPADSMSAQRSENHFNSCSKSSSSHPAFPPVFGSSETMEVDPVDWTMVFAASDSRNIAPRSEKPSARWETKTGLEVEDA
ATCC24261_HD2_protein          SAYANSKHVPQIEPSLAFAIPEPADSMSAQRSENHFNSCSKSSSSHPAFPPVFGSSETMEVDPVDWTMVFAASDSRNIAPRSEKPSARWETKTGLEVEDA
NRRLY17434_HD2_protein         SAYANSKHVPQIEPSLAFAIPEPADSMSAQRSENYFNSCSKSSSSHPSFPPVFGSSETMEVDPVDWTMVFAASDSRNIAPRSEKPSARWETKTGLEVEDA
KBP2604_HD2_protein            SAYANSKHVPQIEPSLAFAIPEPADSMSAQRSENHFNSCSKSSSSHPAFPPVFGSSETMEVDPVDWTMVFAASDSRNIAPRSEKPSARWETKTGLEVEDA
ATCC24201_HD2_protein          SAYANFKHVPQIESSLAFAVPEPADSMSAQCSKNQFNNCSESSSSHPAFPPVFSSSETMEVDPVDWTMVFAATNSRSIAPMYEKPSARWESKTGLEVEDA
ATCC24229_HD2_protein          SAYANFKHVPQIESSLAFAVPEPADSMSAQCSKNQFNNCSESSSSHPAFPPVFSSSETMEVDPVDWTMVFAATNSRSIAPMYEKPSARWESKTGLEVEDA
Clustal Consensus              ***** *:*****.*****:***.:***** *:* :*.**::** *. *****..**** ************::**.***  ********:*.*******
```

```
                                  410       420       430       440
                         ....|....|....|....|....|....|....|....|
CRUB1490_HD2_protein    MLDVSSNMTTGKQTFCEKEG--------------------
CRUB1149_HD2_protein    MLDVSSNMTTGKQTFCEKEG--------------------
CRUB0853_HD2_protein    MLDVSSNMTTGKQTFCEEEG--------------------
GY13L04_HD2_protein     MLDVSPNMTTGK----------------------------
ZP869_HD2_protein       MLDVSPNMTTGK----------------------------
ZP922_HD2_protein       MLDVSPNMTTGK----------------------------
CBS7918_HD2_protein     MLDVNLNMTTGK----------------------------
CBS6938_HD2_protein     MLDVNLNMTTGK----------------------------
ATCC24261_HD2_protein   MLDVNLNMTTGK----------------------------
NRRLY17434_HD2_protein  MLDVNLNMTTGK----------------------------
KBP2604_HD2_protein     MLDVNLNMTTGK----------------------------
ATCC24201_HD2_protein   MIDVSSNTTAGKQTSCEKEGCSHRETPDDIMVRPQFQISF
ATCC24229_HD2_protein   MIDVSSNTTAGKQTSCEKEGCSHRETPDDIMVRPQFQISF
Clustal Consensus       *:**. * *:**
```

Homeodomain determined using Pfam server

Nuclear Localization Signal using SeqNLS with a o.5 cutoff
