# Supplementary material for: Genetic Dissection of Sexual Reproduction in a Primary Homothallic Basidiomycete
Source: PLoS Genet. 2016 Jun 21;12(6):e1006110. doi: 10.1371/journal.pgen.1006110 (PMC4915694; doi:10.1371/journal.pgen.1006110)
Supplement: S4 Fig — E. coli BHT101 transformants expressing various combinations of fusion proteins (C) growing on LB/ X-gal media plates (A).and on MacConkey/maltose medium plates (B). (PDF) [file pgen.1006110.s004.pdf]

A Luria-Bertani media supplemented with: 100µg/ml ampicillin, 50µg/ml kanamycin, 0,5mM IPTG and 40µg/ml X-gal

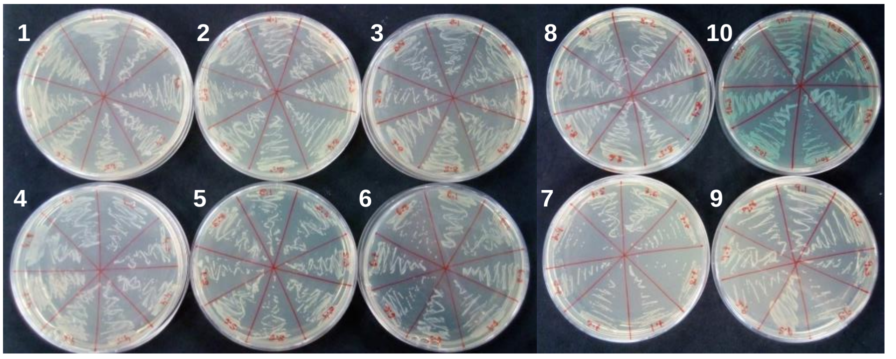

Plates after 24h at 30°C

B MacConkey media supplemented with: 1% Maltose, 100µg/ml ampicillin, 50µg/ml kanamycin and 0,5mM IPTG

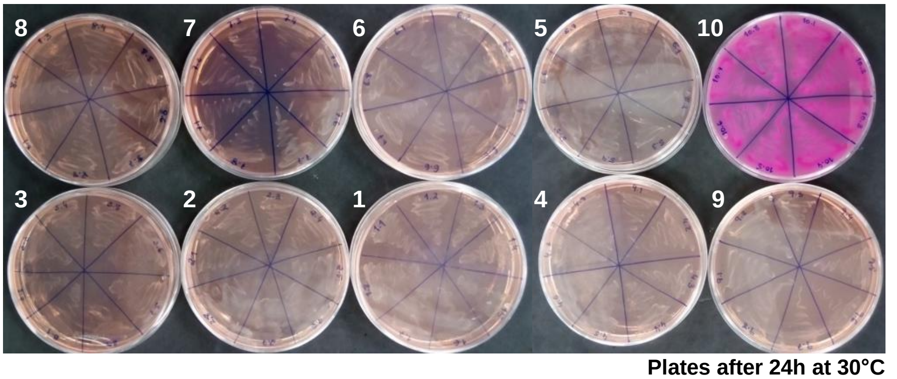

Plates after 24h at 30°C

C

Plate number	***Plasmids present in BTH101 E. coli***	***Testing***
1	*pKNT25+HD1 and pUT18+HD2*	*heterodimerization*
2	*pKNT25+HD2 and pUT18+HD1*	*heterodimerization*
3	*pKNT25+HD1 and pUT18+HD1*	*homodimerization*
4	*pKNT25+HD2 and pUT18+HD2*	*homodimerization*
5	*pKNT25 and pUT18*	*negative control*
6	*pKNT25+HD1 and pUT18*	*negative control*
7	*pKNT25+HD2 and pUT18*	*negative control*
8	*pKNT25 and pUT18+HD1*	*negative control*
9	*pKNT25 and pUT18+HD2*	*negative control*
10	*pKT25-zip and pUT18C-zip*	*positive control*
