# Supplementary material for: Genetic Dissection of Sexual Reproduction in a Primary Homothallic Basidiomycete
Source: PLoS Genet. 2016 Jun 21;12(6):e1006110. doi: 10.1371/journal.pgen.1006110 (PMC4915694; doi:10.1371/journal.pgen.1006110)
Supplement: S5 Fig — A. and B. Three independent diploid strains (X, Y and Z) expressing each of the combinations (D) of fusion proteins (C) growing on selective medium to assess adenine and histidine prototrophy (A) and indicator medium containing X-alpha GAL to assess expression of the MEL1 reporter gene. Individual fusion proteins were unable to activate transcription of the reporter genes (E). (PDF) [file pgen.1006110.s005.pdf]

A Yeast nitrogen base media (without amino acids) supplemented with: 0,02 mg/mL uracil, 2% agar and 2% glucose

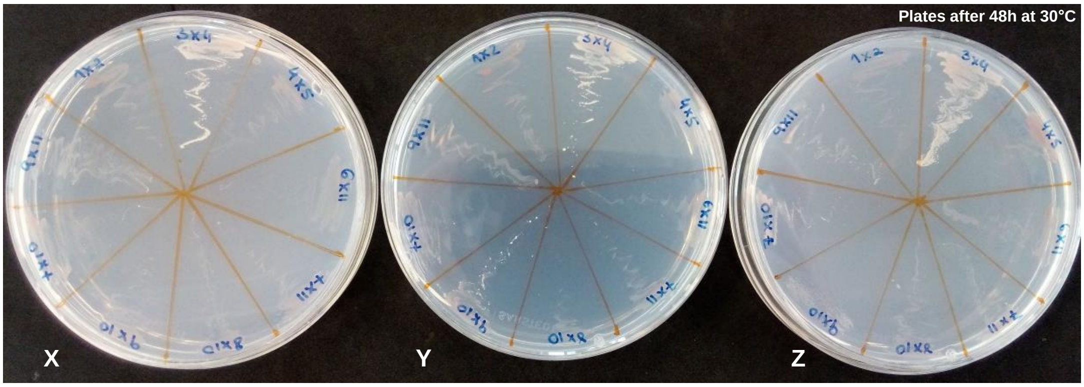

B Yeast nitrogen base media (without amino acids) supplemented with: 0,02 mg/ml uracil, 0,02 mg/ml histidine, 0,02 mg/ml adenine, 2% agar, 2% glucose and 40µg/ml X-α-Gal

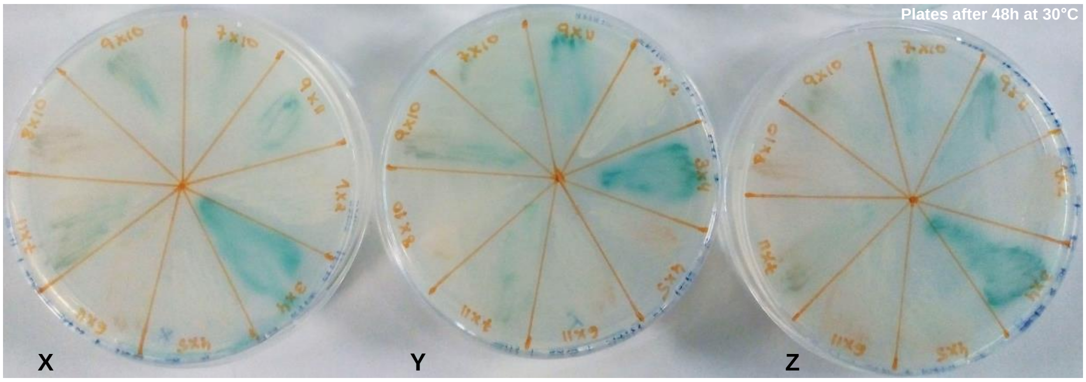

C

Haploid id number	*Plasmid in the haploid S. cerevisiae strain*
1	*pGBKT7 in Y2HGold cells*
2	*pGADT7 in Y187 cells*
3	*pGBKT7-53 in Y2HGold cells*
4	*pGADT7-T in Y187 cells*
5	*pGBKT7-Lam in Y2HGold cells*
6	*pGBKT7+MP181/182 in Y2HGold cells*
7	*pGBKT7+MP181/MP183 in Y2HGold cells*
8	*pGBKT7+MP184/MP185 in Y2HGold cells*
9	*pGBKT7+MP184/MP186 in Y2HGold cells*
10	*pGADT7 +MP187/MP188 in Y187 cells*
11	*pGADT7 +MP189/MP190 in Y187 cells*

D

Mating experiments using the haploid strains	*Objective*
1X2	*Negative control with empty plasmids*
3X4	*Positive control for interaction*
4X5	*Negative control for interaction*
6X11	*Testing for heterodimerization hd1Xhd2*
7X11	*Testing for heterodimerization HD1Xhd2*
8X10	*Testing for heterodimerization hd2Xhd1*
9X10	*Testing for heterodimerization HD2Xhd1*
7X10	*Testing for homodimerization HD1Xhd1*
9X11	*Testing for homodimerization HD2Xhd2*

E

YNB w/o aminoacids +ura+ade+his+leu+X-α-Gal	YNB w/o aminoacids +ura+leu	YNB w/o aminoacids +ura+trp+ade+his+met+X-α-Gal	YNB w/o aminoacids +ura+trp+met

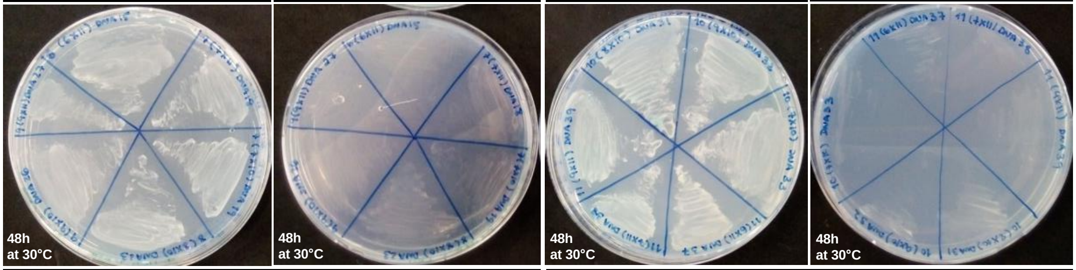

pGBKT7 + HD inserts	pGADT7 + HD inserts
