# Supplementary material for: Genetic Dissection of Sexual Reproduction in a Primary Homothallic Basidiomycete
Source: PLoS Genet. 2016 Jun 21;12(6):e1006110. doi: 10.1371/journal.pgen.1006110 (PMC4915694; doi:10.1371/journal.pgen.1006110)
Supplement: S7 Fig — Autoradiographs of the results obtained for each mutant. Expected fragment sizes are shown. (PDF) [file pgen.1006110.s007.pdf]

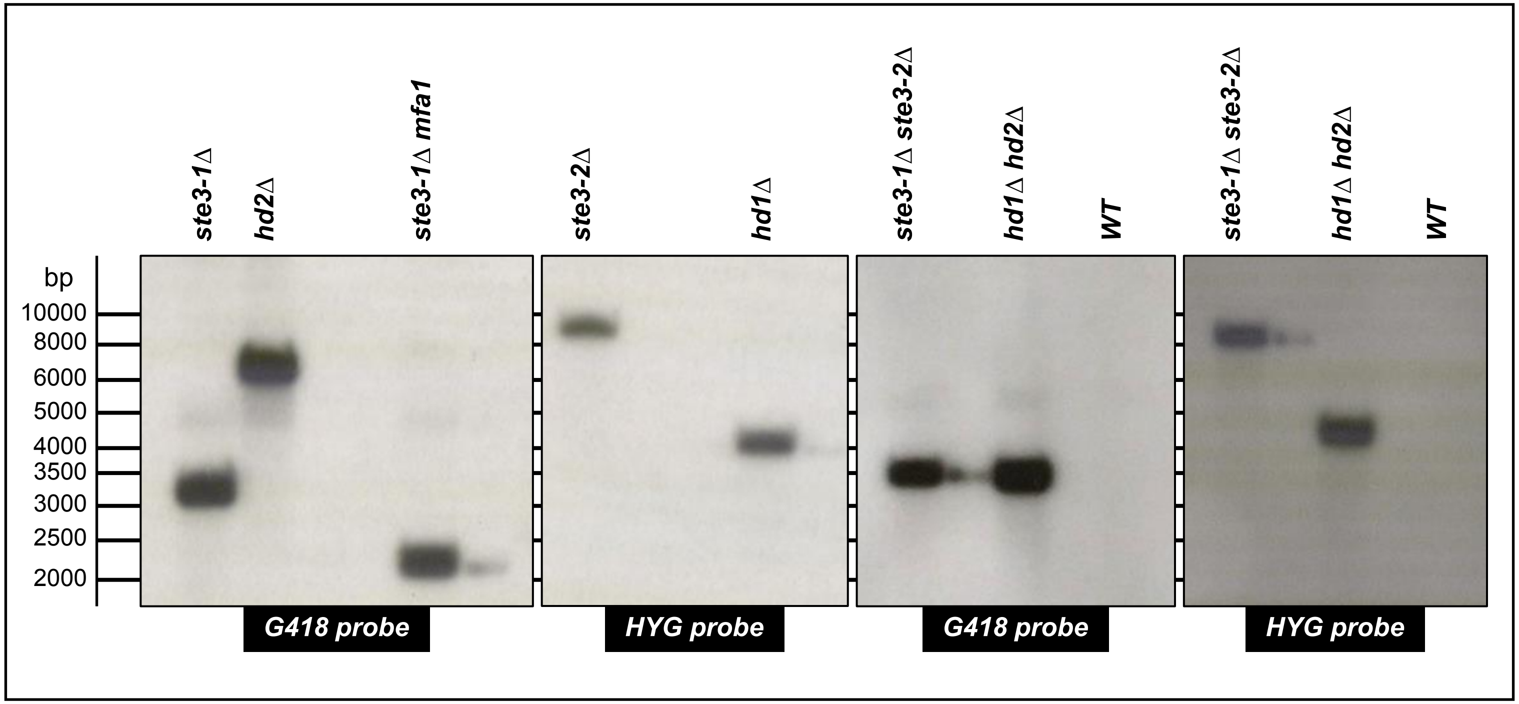

Strains	Relevant genotypes	Expected fragment size / used probe
ste3-1Δ	*ste3-1Δ::G418*	3196 bp / G418
hd2Δ	*hd2Δ::G418*	6651 bp / G418
ste3-1Δ mfa1Δ	*ste3-1/mfa1Δ::G418*	2213 bp / G418
ste3-2Δ	*ste3-2Δ::HYG*	8337 bp / HYG
hd1Δ	*hd1Δ::HYG*	4332 bp / HYG
ste3-1Δ ste3-2Δ	*ste3-2Δ::HYG/ste3-1Δ::G418*	3196 bp / G418; 8337 bp / HYG
hd1Δ hd2Δ	*hd1Δ::HYG/hd2Δ::G418*	3189 bp / G418; 4332 bp / HYG
WT (CBS 6938)		No bands expected with any of the probes
