# Supplementary material for: Genetic Dissection of Sexual Reproduction in a Primary Homothallic Basidiomycete
Source: PLoS Genet. 2016 Jun 21;12(6):e1006110. doi: 10.1371/journal.pgen.1006110 (PMC4915694; doi:10.1371/journal.pgen.1006110)
Supplement: S1 Table — Proteins were aligned using ClustalW as implemented in Bioedit (Hall, 1999). (PDF) [file pgen.1006110.s008.pdf]

S1 Table. Amino acid identity (%) between different alleles of pheromone receptors from selected basidiomycete species. Proteins where aligned using ClustalW as implemented in Bioedit (Hall, 1999).

Species	Mating	Pheromone receptor gene	Accession number / Genome Locus (coordinates)	% aa identity
Phaffia rhodozyma (CRUB 1149)	Homothallic	*STE3-1*	PRJNA307837/ NODE_186 (11928..13388)	49
		STE3-2	PRJNA307837/ NODE_198(2416..3807)	
Phaffia rhodozyma (CBS 6938)	Homothallic	*STE3-1*	CED85384.1	50
		STE3-2	CED85379.1	
Phaffia rhodozyma (CBS 7918)	Homothallic	*STE3-1*	PRJNA306035/ LSVH01000226.1 (11858..13318)	50
		STE3-2	PRJNA306035/ LSVH01000253.1 (7004..8395)	
Microbotryum violaceum	Heterothallic bipolar	*A1*	ABU62847.1	20
		A2	ABU62846.1	
Ustilago hordei	Heterothallic bipolar	*Pra1*	CAJ41875.1	23
		Pra2	AAD56044.1	
Cryptococcus flavescens	Heterothallic tetrapolar	*A2*	CDR19322.1	29
		A1	CDR19327.1	
Sporidiolobos salmonicolor	Heterothallic tetrapolar	*A2*	ADM24775.1	26
		A1	ADM24772.1	
Cryptococcus neoformans	Heterothallic bipolar	*STE3 a*	AAN75624.1	31
		STE3 alpha	AAN75724.1	
Cryptococcus gattii	Heterothallic bipolar	*STE3 a*	AEG78597.1	31
		STE3 alpha	AEG78622.1	
Sporisorium reilianum	Heterothallic tetrapolar	*Pra1*	CAI59749.1	Pra1 vs. Pra2: 23<br>Pra1 vs. Pra3: 28<br>Pra2 vs. Pra3: 24
		Pra2	CBQ71125.1	
		Pra3	ABW21687.1	
