# Supplementary material for: Genetic Dissection of Sexual Reproduction in a Primary Homothallic Basidiomycete
Source: PLoS Genet. 2016 Jun 21;12(6):e1006110. doi: 10.1371/journal.pgen.1006110 (PMC4915694; doi:10.1371/journal.pgen.1006110)
Supplement: S2 Table — (PDF) [file pgen.1006110.s009.pdf]

S2 Table. Data from crosses of double and triple mutant strains.

	Number of basidia per plate		
**Strains**	**Assay 1**	**Assay 2**	**Assay 3**
CBS 6938	*7900**		
ste3-1Δmfa1Δ ***X****ste3-2Δmfa2Δ*	70	38	*21*
ste3-1Δmfa1Δhd1Δ ***X*** *ste3-2Δmfa2Δhd2Δ*	9	0	2

**estimated value*
