# Supplementary material for: Genetic Dissection of Sexual Reproduction in a Primary Homothallic Basidiomycete
Source: PLoS Genet. 2016 Jun 21;12(6):e1006110. doi: 10.1371/journal.pgen.1006110 (PMC4915694; doi:10.1371/journal.pgen.1006110)
Supplement: S3 Table — A. Sporulation data pertaining to plot E (Fig 1), plot C (Fig 3) and plot A (Fig 4). B. Sporulation data pertaining to plot G (Fig 1). (PDF) [file pgen.1006110.s010.pdf]

S3 Table. A) Sporulation data pertaining to plot E (Figure 2), plot C (Figure 3) and plot A (Figure 4).

	Assay	Nº basidia per plate 1	Nº basidia per plate 2	Nº basidia per plate 3	Mean nº of basidia per plate	Mean nº of basidia per plate (trunc.)	Mean	Standard deviation
CBS 6938	1	836	1035	501	790.7	791	905.0	139.1
	2	995	981	1203	1059.7	1060		
	3	621	969	1003	864.3	864		
ste3-1Δ	1	1012	571	620	734.3	734	814.0	92.9
	2	743	851	783	792.3	792		
	3	943	854	950	915.7	916		
ste3-2Δ	1	1231	1153	918	1100.7	1101	1142.7	113.3
	2	1052	920	1842	1271.3	1271		
	3	820	998	1351	1056.3	1056		
hd2Δ	1	8	23	15	15.3	15	13.7	2.3
	2	4	12	16	10.7	11		
	3	20	13	13	15.3	15		
spo11Δ	1	587	387	720	564.7	565	649.3	77.6
	2	503	881	610	664.7	665		
	3	532	689	932	717.7	718		

S3 Table. B) Sporulation data pertaining to plot G (Figure 2).

	Assay	Nº basidia per plate 1	Nº basidia per plate 2	Nº basidia per plate 3	Mean nº of basidia per plate	Mean nº of basidia per plate (trunc.)	Mean	Standard deviation
CBS 6938	1	904	832	861	865.7	866	897.3	36.2
	2	828	903	937	889.3	889		
	3	809	1004	998	937	937		
ste3-1Δ /mfa2Δ	1	854	743	792	796.3	796	881.7	76.5
	2	950	986	893	943	943		
	3	991	701	1027	906.3	906		
ste3-2Δ /mfa1Δ	1	725	1136	1021	960.7	961	1004.3	38.9
	2	1118	1002	928	1016	1016		
	3	915	1203	989	1035.7	1036		
hd1Δhd2Δ+HD1	1	39	32	22	30.7	31	43.7	11.4
	2	42	41	59	47.3	47		
	3	53	60	47	53.3	53		
