# Supplementary material for: Genetic Dissection of Sexual Reproduction in a Primary Homothallic Basidiomycete
Source: PLoS Genet. 2016 Jun 21;12(6):e1006110. doi: 10.1371/journal.pgen.1006110 (PMC4915694; doi:10.1371/journal.pgen.1006110)
Supplement: S4 Table — (PDF) [file pgen.1006110.s011.pdf]

S4 Table. List of plasmids and primers used in the Matchmaker® Gold Yeast Two-Hybrid System.

Plasmids	Relevant features
pGBKT7 DNA-BD	*plasmid encoding the yeast Gal4 DNA-binding domain*
pGADT7 AD	*plasmid encoding the yeast Gal4 activation domain*
pGBKT7-53	*Plasmid use for positive control of protein interaction*
pGADT7-T	*Plasmid use for positive control of protein interaction*
pGBKT7-Lam	*Plasmid use for negative control of protein interaction*
pGBKT7+MP181-MP182	*pGBKT7 plasmid containing HD1 gene (short version)*[1] *cloned in frame with the GAL4 DNA-BD*
pGBKT7+MP181-MP183	*pGBKT7 plasmid containing HD1 gene cloned in frame with the GAL4 DNA-BD*
pGBKT7+MP184-MP185	*pGBKT7 plasmid containing HD2 gene (short version)*[1] *cloned in frame with the GAL4 DNA-BD*
pGBKT7+MP184-MP186	*pGBKT7 plasmid containing HD2 gene cloned in frame with the GAL4 DNA-BD*
pGADT7+MP187-MP188	*pGADT7plasmid containing HD1 gene (short version)*[1] *cloned in frame with the GAL4 activation domain*
pGADT7+MP189-MP190	*pGADT7plasmid containing HD2 gene (short version)*[1] *cloned in frame with the GAL4 activation domain*

Primers (5'-3')	Relevant information
MP181- CATCATGGAGGAGCAGAAGCTGATCTCAGAGGAGGACCTGATGGAACCGAGTCCGGCG	*Amplifies fragment comprising the complete N-terminal and homeodomain regions of the HD1 gene plus a tail of 40 bp at both 5'*[2] *and 3'*[3] *ends (complementary to the pGBKT7 plasmid)*
MP182- TCAAGACCCGTTTAGAGGCCCCAAGGGGTTATGCTAGTTACATCCAGCCGCTGCGACG	
MP183- TCAAGACCCGTTTAGAGGCCCCAAGGGGTTATGCTAGTTATTAGCAGTCGAATACTTCGCCC	*With MP181 primer, amplifies fragment comprising the complete HD1 gene plus a tail of 40 bp at both 5'*[2] *and 3'*[3] *ends (complementary to the pGBKT7 plasmid)*
MP184- CATCATGGAGGAGCAGAAGCTGATCTCAGAGGAGGACCTGATGTATAGCTTCACCAAGTTGCC	*Amplifies fragment comprising the complete N-terminal and homeodomain regions of the HD2 gene plus a tail of 40 bp at both 5'*[2] *and 3'*[3] *ends (complementary to the pGBKT7 plasmid)*
MP185- TCAAGACCCGTTTAGAGGCCCCAAGGGGTTATGCTAGTTAAGAGCGCCGATTACGGCG	
MP186- TCAAGACCCGTTTAGAGGCCCCAAGGGGTTATGCTAGTTATTATTTCCCCGTTGTCATGTTCAGG	*With MP184 primer, amplifies fragment comprising the complete HD2 gene plus a tail of 40 bp at both 5'*[2] *and 3'*[3] *ends (complementary to the pGBKT7 plasmid)*
MP187- CGCCGCCATGGAGTACCCATACGACGTACCAGATTACGCTATGGAACCGAGTCCGGCG	*Amplifies fragment comprising the complete N-terminal and homeodomain regions of the HD1 gene plus a tail of 40 bp at both 5'*[4] *and 3'*[5] *ends (complementary to the pGADT7 plasmid)*
MP188- TTGAAGTGAACTTGCGGGGTTTTTCAGTATCTACGATTCACATCCAGCCGCTGCGACG	
MP189- CGCCGCCATGGAGTACCCATACGACGTACCAGATTACGCTATGTATAGCTTCACCAAGTTGCC	*Amplifies fragment comprising the complete N-terminal and homeodomain regions of the HD2 gene plus a tail of 40 bp at both 5'*[4] *and 3'*[5] *ends (complementary to the pGADT7 plasmid)*
MP190- TTGAAGTGAACTTGCGGGGTTTTTCAGTATCTACGATTCAAGAGCGCCGATTACGGCG	

[1]*Short version of the genes HD1 and HD2 comprises the complete N-terminal region and homeodomain region of each of the genes (first 183 amino acids of HD1 and the first 196 amino acids of HD2).*
[2]*5'-tail before MCS in pGBKT7 plasmid: CATCATGGAGGAGCAGAAGCTGATCTCAGAGGAGGACCTG*
[3]*3'-tail after MCS in pGBKT7 p plasmid: TCAAGACCCGTTTAGAGGCCCCAAGGGGTTATGCTAGTTA*
[4]*5'-tail before MCS in pGADT7 plasmid: CGCCGCCATGGAGTACCCATACGACGTACCAGATTACGCT*
[5]*3'-tail after MCS in pGADT7 plasmid: TTGAAGTGAACTTGCGGGGTTTTTCAGTATCTACGATTCA*
