# Supplementary material for: Genetic Dissection of Sexual Reproduction in a Primary Homothallic Basidiomycete
Source: PLoS Genet. 2016 Jun 21;12(6):e1006110. doi: 10.1371/journal.pgen.1006110 (PMC4915694; doi:10.1371/journal.pgen.1006110)
Supplement: S6 Table — (PDF) [file pgen.1006110.s013.pdf]

S6 Table. List of strains used in bacterial and yeast two hybrid assays.

Strains	Relevant features
DH5α	*Escherichia coli laboratory strain (Gibco-BRL, Carlsbad, CA, USA)*
BTH101	*Escherichia coli reporter strain for BACTH (Bacterial Adenylate Cyclase Two-Hybrid System Kit) from EUROMEDEX (Cat. No. EUK001)*
Y2HGold	*Saccharomyces cerevisiae host strain for Matchmaker Gold Yeast Two-Hybrid System from Clontech (Cat. No. 630489); MATa, trp1-901, leu2-3, 112,ura3-52, his3-200, gal4Δ, gal80Δ,LYS2 : : GAL1UAS–Gal1TATA–His3, GAL2UAS–Gal2TATA–Ade2, URA3 : : MEL1UAS–Mel1TATA, AUR1-C, MEL1*
Y187	*Saccharomyces cerevisiae host strain for Matchmaker Gold Yeast Two-Hybrid System from Clontech (Cat. No. 630489); MATα, ura3-52, his3-200, ade2-101, trp1-901, leu2-3, 112, gal4Δ, gal80Δ, met–,URA3 : : GAL1UAS–Gal1TATA–LacZ,MEL1*
