# Supplementary material for: Genetic Dissection of Sexual Reproduction in a Primary Homothallic Basidiomycete
Source: PLoS Genet. 2016 Jun 21;12(6):e1006110. doi: 10.1371/journal.pgen.1006110 (PMC4915694; doi:10.1371/journal.pgen.1006110)
Supplement: S7 Table — (PDF) [file pgen.1006110.s014.pdf]

S7 Table. Primers and plasmids used for the construction of all gene deletion fragments except for gene STE3-2.

		Primers use to construct gene deletion fragments						Primers used for mutant confirmation		
**Gene or region to knockout**	**Backbone plasmid**	**Primers upstream from gene (5'-3')**	**Amplified fragment (bp)**	**Primers downstream from gene (5'-3')**	**Amplified fragment (bp)**	**Nested primers (5'-3')**	**Amplified fragment (bp)**	**Primers (5'-3')**	**Amplified fragment (bp)**	**Description**
Ste3-1	pPR2TN	MP066 (Kpn I) - GCGTCGGTACCGATGGAATGAGAGAG<br>MP067 (EcoR I) - CTGGCGAATTCTGCTGTCTTTTAAGTTC	848	MP064 (Not I) - AGTGAGCGGCCGCATGATGCGCGCGACG<br>MP065 (Hind III) - GTTGCAAGCTTCAATCTTAGCTGCGCTGG	1128	MP068 - GCGCTGGACAACACGGAGC<br>MP069 - CAGTTTTTCATTCAATCCGAACGG	3869	MP029 - CGGTGGGCGTTCTGGTCGGAC<br>MP030 - ACTCTGATGGCGAAGCAACGGC	836	Amplification of the *Ste3-1* gene (partial)
								MP067 - CTGGCGAATTCTGCTGTCTTTTAAGTTC<br>MP092 - TTCCACCATGATATTCGGCAAGCAGG	2220	Upstream flanking region of *Ste3-1* gene and partial resistance cassette
								MP091 - AAGATGGATTGCACGCAGGTTCTCC<br>MP065 - GTTGCAAGCTTCAATCTTAGCTGCGCTGG	2500	Downstream flanking region of *Ste3-1* gene and partial resistance cassette
Hd1	pBS-HYG	MP072 (Cla I) - AGATGATCGATTTTTCTATCTGGTGC<br>MP073 (Kpn I) - ATGATGGTACCCGTAAGCTGTAGTTTC	775	MP070 (Sac I) - CTGTCGAGCTCAAACCGCTCTCAACC<br>MP071 (Not I) - GGTTTGCGGCCGCTAGATTTCCCTTTTG	913	MP074 - AACGAAAGATGAAGACACCAG<br>MP075 - GTTTCACCAGAACATATCAGG	3508	MP083 - AAAACCTCTCCAAGCGCC<br>MP084 - TCTACATCGGTCTCTTCC<br>MP154- ATGAGATCGTTCAACAGC (paired withMP086)	1002<br>2580	Amplification of the *Hd1* gene (partial)
								MP060 - GTGGAAACCGACGCCCCAGC<br>MP073 - ATGATGGTACCCGTAAGCTGTAGTTTC	1500	Upstream flanking region of *Hd1* gene and partial resistance cassette
								MP070 - CTGTCGAGCTCAAACCGCTCTCAACC<br>MP063 - TTTGCCCTCGGACGAGTGCTGG	2932	Downstream flanking region of *Hd1* gene and partial resistance cassette
Hd2	pPR2TN	MP076 (EcoR I) - GATGGGAATTCTTTCTATCTGGTGCAC<br>MP073 (Kpn I) - ATGATGGTACCCGTAAGCTGTAGTTTC	774	MP077 (Not I) - GACGAGCGGCCGCTAAACCTCTTGTGAG<br>MP078 (Hind III) - ACTCCAAGCTTTCTCTATGGTTGAGAG	629	MP079 - TTTCTATCTGGTGCACAACTTC<br>MP080 - TATGGTTGAGAGTAATGAGC	3305	MP085 - TGTACAGTTTCACGAAGC<br>MP086 - GTCTTCCGTTCTTTCTCG<br>MP155 - ACATTTCATTAAGGCTGG (paired withMP085)	916<br>1595	Amplification of the *Hd2* gene (partial)
								MP076 - GATGGGAATTCTTTCTATCTGGTGCAC<br>MP048 - GTACAGTCGCACAACCGACACGC	2254	Upstream flanking region of *Hd2* gene and partial resistance cassette
								MP037 - GCGTGTCGGTTGTGCGACTGTAC<br>MP078 - ACTCCAAGCTTTCTCTATGGTTGAGAG	850	Downstream flanking region of *Hd2* gene and partial resistance cassette
	pJET1.2+Zeo	MP156 (Bsm I) - TTATTGAATGCTTTCTATCTGGTGCAC<br>MP157 (Cla I) - AAAAAATCGATCGTAAGCTGTAGTTTC	776	MP158 (Pst I) - TTATACTGCAGTCTCTATGGTTGAGAGTAATGAGC<br>MP077 (Not I) - GACGAGCGGCCGCTAAACCTCTTGTGAG	630	MP079 - TTTCTATCTGGTGCACAACTTC<br>MP080 - TATGGTTGAGAGTAATGAGC	3305	MP085 - TGTACAGTTTCACGAAGC<br>MP086 - GTCTTCCGTTCTTTCTCG	916	Amplification of the *Hd2* gene (partial)
								MP049 - AGATAGAAACCCAACACTCGC<br>MP114 - ATAAAAGATCTATGGCCAAGTTGACC	1745	Upstream flanking region of *Hd2* gene and partial resistance cassette
								MP115 - ATAATTCTAGATCAGTCCTGCTCCTC<br>MP159 - TTATCTCGGTCAACTGCCGG	1446	Downstream flanking region of *Hd2* gene and partial resistance cassette
Ste3-1/mfa1	pPR2TN	MP087 (Kpn I) - TCATTGGTACCGTGGTAAGTCTGCCTG<br>MP088 (Eco RI) - TCACCGAATTCCACTTGCGCGCTTGC	1058	MP064 (Not I) - AGTGAGCGGCCGCATGATGCGCGCGACG<br>MP065 (Hind III) - GTTGCAAGCTTCAATCTTAGCTGCGCTGG	1126	MP068 - GCGCTGGACAACACGGAGC<br>MP089 - GCTTGCATTCATACATACCTCAGC	4591	MP029 - CGGTGGGCGTTCTGGTCGGAC<br>MP030 - ACTCTGATGGCGAAGCAACGGC	836	Amplification of the *Ste3-1* gene (partial)
								MP100 - TCCATCCTCAACTGATTGC<br>MP101 - AGTGTAAAGAAGTCTCGG	357	Amplification of the *Mfa1* (complete) region
								MP091 - AAGATGGATTGCACGCAGGTTCTCC<br>MP065 - GTTGCAAGCTTCAATCTTAGCTGCGCTGG	2500	Downstream flanking region of *Ste3-1* gene and partial resistance cassette
								MP092 - TTCCACCATGATATTCGGCAAGCAGG<br>MP088 - TCACCGAATTCCACTTGCGCGCTTGC	2430	Downstream flanking region of *Mfa1* gene and partial resistance cassette
Ste3-2/mfa2	pPR2TN	MP093 (EcoR I) - TTTCAGAATTCCACCACATTCTCCCG<br>MP094 (Kpn I) - AGAATTGGTACCCTCGACGGCTTTAGC	824	MP095 - AATATAAGCGGCCGCTACCCTGATACCTTG<br>MP096 -TTCTGAAGCTTAAGTTCTGATTATCTAGAACCATCC	848	MP097 - CTCTACATAGAACTAGAATGGTCC<br>MP098 - GAGTCCTTGATGTTGACGTAACC	3224	MP035 - TTATGCATCAACCGGCGTCTGGC<br>MP036 - GGACACAGAGGCAACRGTAGTTCC	929	Amplification of the *Ste3-2* gene (partial)
								MP102 - TATATCATCTCTCGACCC<br>MP103 - TTCATCTTGTCAGACAGC	611	Amplification of the *Mfa2* (complete) region
								MP091 - AAGATGGATTGCACGCAGGTTCTCC<br>MP099 - ACATGCTGTAGGCTTATCGATAGCG	2400	Downstream flanking region of *Ste3-2* gene and partial resistance cassette
								MP093 - TTTCAGAATTCCACCACATTCTCCCG<br>MP092 - TTCCACCATGATATTCGGCAAGCAGG	2000	Downstream flanking region of *Mfa2* gene and partial resistance cassette
Spo11	pBS-HYG	MP126 (Not I) - TATTTGCGGCCGCGTTGAATGTTTCAGG<br>MP127 (Sac I) - TATTTGAGCTCGCTTTGTCAACCTATGCG	732	MP124 (Kpn I) - TATTTGGTACCTCAAGGAAACATGCG<br>MP125 (Cla I) - TTAATATCGATGTCATTCCCCGAAGC	565	MP128 - AAGGAAACATGCGGGACG<br>MP129 - GCTTTGTCAACCTATGCG	4000	MP130 - TTACTTCCATTCCTTCGGC<br>MP131 - TGACTTGTCCAGTATCTCC	2750	Amplification of the *Spo11* gene (partial)
								MP063 - TTTGCCCTCGGACGAGTGCTGG<br>MP133 - TTGCCGGAGGTGGTTGGATGG	2293	Upstream flanking region of *Spo11* gene and partial resistance cassette
								MP132 - TCGTTCCTCAGCCCTAAGACATGC<br>MP060 - GTGGAAACCGACGCCCCAGC	1344	Downstream flanking region of *Spo11* gene and partial resistance cassette
Mfa1	pJET1.2+Zeo	MP171 (Pst I) - TTTTTCTGCAGACTAGGTACATCACTCG<br>MP 172 (Not I) - TTTATGCGGCCGCTCTTTACACTTTTGC	1165	MP173 (Cla I) - ATTTTATCGATAGGCAATCAGTTGAGG<br>MP174 (Pme I) - TATTAGTTTAAACATAAGGCAGTCGACG	1024	MP175 - ACATCACTCGGTCAGATCC<br>MP176 - ATAAGGCAGTCGACGAGG	3363	MP100 - TCCATCCTCAACTGATTGC<br>MP101 - AGTGTAAAGAAGTCTCGG	357	Amplification of the *Mfa1* (complete) region
								MP171 - TTTTTCTGCAGACTAGGTACATCACTCG<br>MP115 - ATAATTCTAGATCAGTCCTGCTCCTC	1937	Downstream flanking region of *Mfa1* gene and partial resistance cassette
								MP114 - ATAAAAGATCTATGGCCAAGTTGACC<br>MP174 - TATTAGTTTAAACATAAGGCAGTCGACG	1794	Upstream flanking region of *Mfa1* gene and partial resistance cassette
Mfa2	pJET1.2+Zeo	MP162 (Pst I) – TTTTTCTGCAGGATCAGAGGAGTACGATCG<br>MP163 (Not I) - TTTTTGCGGCCGCATGGGATCTTCAGGTAG	998	MP166 (Cla I) - TTTTTATCGATGTATATACGAGATCACC<br>MP167 (Bsm I) - TTTTTGAATGCAGAACGACTGGAAAGAG	1014	MP168 - ATCAGAGGAGTACGATCG<br>MP170 - AGAACGACTGGAAAGAGC	3193	MP102 - TATATCATCTCTCGACCC<br>MP103 - TTCATCTTGTCAGACAGC	611	Amplification of the *Mfa2* (complete) region
								MP162 - TTTTTCTGCAGGATCAGAGGAGTACGATCG<br>MP115 - ATAATTCTAGATCAGTCCTGCTCCTC	1770	Upstream flanking region of *Mfa2* gene and partial resistance cassette
								MP114 - ATAAAAGATCTATGGCCAAGTTGACC<br>MP167 - TTTTTGAATGCAGAACGACTGGAAAGAG	1784	Downstream flanking region of *Mfa2* gene and partial resistance cassette
