# Supplementary material for: Genetic Dissection of Sexual Reproduction in a Primary Homothallic Basidiomycete
Source: PLoS Genet. 2016 Jun 21;12(6):e1006110. doi: 10.1371/journal.pgen.1006110 (PMC4915694; doi:10.1371/journal.pgen.1006110)
Supplement: S8 Table — (PDF) [file pgen.1006110.s015.pdf]

S8 Table. Primers and plasmid used for the construction of the STE3-2 deletion fragment by overlap extension PCR.

Gene or region to knockout	Plasmid	Primers upstream from gene (5'-3')	Amplified fragment (bp)	Primers for HYG resistance cassette (5'-3')	Amplified fragment (bp)	Primers downstream from gene (5'-3')	Amplified fragment (bp)	Nested primers (5'-3')	Amplified fragment (bp)	Primers (5'-3') used for mutant confirmation and amplification description	Amplified fragment (bp)
Ste3-2	pJet1.2<br><br>resistance cassette storage	MP039 - TGTCAGGGCAAAAAGAGAAAAACAAGGGG<br>MP040 - AAGAGCTTGTGTCGGATGAACTGTCGGCTGA TGAGCCGATGATCAGAGGAGTACGATCGAAAGAGAAGAT<br><br>(Amplified from CBS 6938 gDNA)	1165	MP041 - TCGATATATAATCTTCTCTTTCGATCGTACTCCTC TGATCATCGGCTCATCAGCCGACAGTTCATCCGAC<br>MP042 - TATACTACAACATTACCGAAGGTATCAAGGTATC AGGGTAATCATGAGAGATGACGGAGATGATGGTGATG<br><br>(Amplified from pBS-HYG plasmid)	1899	MP043 - TCTGTTGACCATCACCATCATCTCCGTCATCTCT CATGATTACCCTGATACCTTGATACCTTCGGTAATG<br>MP044 – AACGAGCAAGGAGACAAACAACAGGTCGAG<br><br>(Amplified from CBS 6938 gDNA)	609	MP045 - GAGAAAAACAAGGGGAGATTTGGG<br>MP046 - ACAGGTCGAGGGCAGCAAGGG	3480	MP035 - TTATGCATCAACCGGCGTCTGGC<br>MP036 - GGACACAGAGGCAACRGTAGTTCC<br>(Amplification of the *Ste3-2* gene (partial))	929
										MP060 - GTGGAAACCGACGCCCCAGC<br>MP061 - GAGGGCTGTTGACGATAGGG<br>(Downstream flanking region of *Ste3-2* gene and partial resistance cassette)	1156
										MP063 - TTTGCCCTCGGACGAGTGCTGG<br>MP039 - TGTCAGGGCAAAAAGAGAAAAACAAGGGG<br>(Upstream flanking region of *Ste3-2* gene and partial resistance cassette)	2542
