# Supplementary material for: Genetic Dissection of Sexual Reproduction in a Primary Homothallic Basidiomycete
Source: PLoS Genet. 2016 Jun 21;12(6):e1006110. doi: 10.1371/journal.pgen.1006110 (PMC4915694; doi:10.1371/journal.pgen.1006110)
Supplement: S9 Table — (PDF) [file pgen.1006110.s016.pdf]

S9 Table. Primers and plasmid used for the construction of the Zeocin resistance cassette.

**Resistance cassette**	**Backbone Plasmid**	**Primers for promoter (*Ptef*) (5'-3')**	**Amplified fragment (bp)**	**Primers for *Sh ble* gene (5'-3')**	**Amplified fragment (bp)**	**Primers for terminator (*Tgpd*) (5'-3')**	**Amplified fragment (bp)**	**Nested primers (5'-3')**	**Amplified fragment (bp)**	**Other information**
Zeocin	pJET1.2	MP112 (Not I) - ATAATGCGGCCGCATCGGCTCATCAGC<br>MP113 (Bgl II) - ATGCGAGATCTGGTGAAGCTGTTCGAG<br><br>(Amplified from CBS 6938 gDNA)	398	MP114 (Bgl II) - ATAAAAGATCTATGGCCAAGTTGACC<br>MP115 (Xba I) - ATAATTCTAGATCAGTCCTGCTCCTC<br><br>(Amplified from pcDNA-3.1/ZEO plasmid from Invitrogen)	374	MP116 (Xba I) - ATAATTCTAGAAGGCCTACGGTTCTCTCC<br>MP117 (Cla I) - GACTGATCGATATCATGAGAGATGACGG<br><br>(Amplified from CBS 6938 gDNA)	396	MP118 - ATCGGCTCATCAGCCGACAGTTCATC<br>MP119 - ATCATGAGAGATGACGGAGATGATGG	1175	Primers MP118/MP119 were used to sequence the complete resistance cassette
