# Supplementary material for: Genetic Dissection of Sexual Reproduction in a Primary Homothallic Basidiomycete
Source: PLoS Genet. 2016 Jun 21;12(6):e1006110. doi: 10.1371/journal.pgen.1006110 (PMC4915694; doi:10.1371/journal.pgen.1006110)
Supplement: S10 Table — (PDF) [file pgen.1006110.s017.pdf]

S10 Table. Primers and plasmid used for the construction of the complementation constructs.

**Complemented gene**	**Backbone Plasmid**	**Primers (5'-3')**	**Amplified fragment (bp)**	Description of amplified region	complementation plasmid	Linearization of complementation plasmid	Mutant strain transformed with complementation plasmid	**Primers used for mutant confirmation (5'-3')**	**Amplified fragment (bp)**	**Description**
MFA2	pUC18+rDNA+ZEO	MP134 (Pst I) – ATTATCTGCAGACCTCGAGACCCAGCTCCG<br>MP135 (BamH I) - ATTATGGATCCAGTGGTCGCCAAAGCTGACG	1122	MFA gene plus 500bp upstream and downstream of the gene	pUC18+rDNA+ZEO+MP134/MP135	Restriction enzyme Cla I	CBS 6938_ste3-2/mfa2Δ	MP102 - TATATCATCTCTCGACCC<br>MP103 - TTCATCTTGTCAGACAGC	611	Amplification of the *MFA2* (complete) region
STE3-1	pUC18+rDNA+ZEO	MP136 (Pst I) - TATTACTGCAGTGTATCTCAAGTCTTGCC<br>MP137 (BamHI) - TATTAGGATCCTTTCCGAGATTGATGGTCG	1994	STE3-1 gene plus 300bp upstream and downstream of the gene	pUC18+rDNA+ZEO+MP136/MP137		CBS 6938_ste3-1/ste3-2Δ	MP029 - CGGTGGGCGTTCTGGTCGGAC<br>MP030 - ACTCTGATGGCGAAGCAACGGC	836	Amplification of the *STE3-1* gene (partial)
HD1	pUC18+rDNA+ZEO	MP138 (Pst I) - AAAAACTGCAGGAAAGATAGTGAAACTGG<br>MP139 (BamH I) - AATTTGGATCCTTTCATCGGGCTCAAGC	2459	HD1 gene plus 300bp upstream and downstream of the gene	pUC18+rDNA+ZEO+MP138/MP139		CBS 6938_hd1/hd2Δ	MP083 - AAAACCTCTCCAAGCGCC<br>MP084 - TCTACATCGGTCTCTTCC	1002	Amplification of the *HD1* gene (partial)
							CBS 6938_hd1Δ	MP083 - AAAACCTCTCCAAGCGCC<br>MP084 - TCTACATCGGTCTCTTCC	1002	Amplification of the *HD1* gene (partial)
