# Supplementary material for: Genetic Dissection of Sexual Reproduction in a Primary Homothallic Basidiomycete
Source: PLoS Genet. 2016 Jun 21;12(6):e1006110. doi: 10.1371/journal.pgen.1006110 (PMC4915694; doi:10.1371/journal.pgen.1006110)
Supplement: S11 Table — (PDF) [file pgen.1006110.s018.pdf]

S11 Table. List of plasmids and primers used in the Bacterial Adenylate Cyclase Two-Hybrid System.

Plasmids	Relevant features
pUT18	*High copy number vector that encodes the T18 fragment that is fused downstream of the MCS. This vector allows creating in-frame fusions at the N-terminal end of T18. BACTH System Kit from EUROMEDEX*
pKNT25	*Low copy number vector that encodes the T25 fragment that is fused in frame downstream from a MCS. This allows creating in-frame fusions at the N-terminal end of T25. BACTH System Kit from EUROMEDEX*
pUT18C-ZIP	*The plasmids pKT25-zip and pUT18C-zip serve as positive controls for complementation in the BACTH System Kit from EUROMEDEX. When pKT25-zip and pUT18C-zip are co-transformed into BTH101 E. coli cells, they restore a Cya+ phenotype. Vector pKT25-zip has a leucine zipper genetically fused in frame to the T25 fragment, while pUT18C-zi has a leucine zipper genetically fused in frame to the T18 fragment.*
pKNT25-ZIP	
pEX-K4-HD1	*Eurofins in-house standard vector with HD1 synthetic gene*
pEX-K4-HD2	*Eurofins in-house standard vector with HD2 synthetic gene*
pKNT25+HD1	*Plasmid with a HD1 in frame fusion at the N-terminal end of T25 subunit*
pKNT25+HD2	*Plasmid with a HD2 in frame fusion at the N-terminal end of T25 subunit*
pUT18+HD1	*Plasmid with a HD1 in frame fusion at the N-terminal end of T18 subunit*
pUT18+HD2	*Plasmid with a HD2 in frame fusion at the N-terminal end of T18 subunit*
**Primers (5'-3')**	**Relevant information**
MP177(Hind III) - ACGCCAAGCTTGATGGAACCGAGTCCG	*MP177-MP178 amplify a 1543 bp fragment containing HD1 synthesized gene present on plasmid pEX-K4-HD1, with restriction sites for Hind III and Pst I in the fragment extremities*
MP178 (Pst I) - TCGACCTGCAGGCGCAGTCGAATACTTC	
MP179 (Hind III) - ACGCCAAGCTTGATGTATAGCTTCACC	*MP179-MP180 amplify a 1261 bp fragment containing HD2 synthesized gene present on plasmid pEX-K4-HD2, with restriction sites for Hind III and Pst I in the fragment extremities.*
MP180 (Pst I) - TCGACCTGCAGGCTTTCCCCGTTGTCATG	
MP191 - ACACTTTATGCTTCCGGC	*Allow the amplification and sequencing of the constructions performed in plasmid pKNT25.*<br>*Fragment MP191-MP192 amplified in:*<br>*- empty pKNT25 plasmid: 176bp*<br>*- pKNT25+HD1:1694bp*<br>*- pKNT25+HD2:1412bp*
MP192 - ACCAGCCTGATGCGATTGC	
MP193 - TGGCACGACAGGTTTCCC	*Allow the amplification and sequencing of the constructions performed in plasmid pUT18.*<br>*Fragment MP193-MP194 amplified in:*<br>*- empty pUT18 plasmid: 284 bp*<br>*- pUT18+HD1: 1802 bp*<br>*- pUT18+HD2: 1520 bp*
MP194 - ACAAGTCGATGCGTTCGC	
