# Supplementary material for: Genetic Dissection of Sexual Reproduction in a Primary Homothallic Basidiomycete
Source: PLoS Genet. 2016 Jun 21;12(6):e1006110. doi: 10.1371/journal.pgen.1006110 (PMC4915694; doi:10.1371/journal.pgen.1006110)
Supplement: S12 Table — (PDF) [file pgen.1006110.s019.pdf]

S12 Table. Accession numbers for all sequences obtained and used in this work.

Strain	*Gene Sequence*	Accession number / Scaffold (coordinates)
Phaffia rhodozyma KBP 2604	*STE3-1 (partial)*	*KU315740*
Phaffia rhodozyma ATCC 24261	*STE3-1 (partial)*	*KU315745*
Phaffia rhodozyma NRRL Y-17434	*STE3-1 (partial)*	*KU315746*
Phaffia rhodozyma GY13L04	*STE3-1 (partial)*	*KU315744*
Phaffia rhodozyma CRUB 1151	*STE3-1 (partial)*	*KU315742*
Phaffia rhodozyma CRUB 0853	*STE3-1 (partial)*	*KU315743*
Phaffia rhodozyma CRUB 1490	*STE3-1 (partial)*	*KU315741*
Phaffia rhodozyma ATCC 24229	*STE3-1 (partial)*	*KU315749*
Phaffia rhodozyma ATCC 24201	*STE3-1 (partial)*	*KU315750*
Phaffia rhodozyma ZP 922	*STE3-1 (partial)*	*KU315747*
Phaffia rhodozyma ZP 869	*STE3-1 (partial)*	*KU315748*
Phaffia rhodozyma CBS 6938	*STE3-1*	*CED85384.1*
Phaffia rhodozyma CBS 7918	*STE3-1*	*PRJNA306035/ LSVH01000226.1 (11858..13318)*
Phaffia rhodozyma CRUB 1149	*STE3-1*	*PRJNA307837/ NODE_186 (11928..13388)*
Phaffia rhodozyma KBP 2604	*STE3-2 (partial)*	*KU315756*
Phaffia rhodozyma ATCC 24261	*STE3-2 (partial)*	*KU315759*
Phaffia rhodozyma NRRL Y-17434	*STE3-2 (partial)*	*KU315754*
Phaffia rhodozyma GY13L04	*STE3-2 (partial)*	*KU315753*
Phaffia rhodozyma CRUB 1151	*STE3-2 (partial)*	*KU315755*
Phaffia rhodozyma CRUB 0853	*STE3-2 (partial)*	*KU315757*
Phaffia rhodozyma CRUB 1490	*STE3-2 (partial)*	*KU315760*
Phaffia rhodozyma ATCC 24229	*STE3-2 (partial)*	*KU315758*
Phaffia rhodozyma ATCC 24201	*STE3-2 (partial)*	*KU315761*
Phaffia rhodozyma ZP 922	*STE3-2 (partial)*	*KU315752*
Phaffia rhodozyma ZP 869	*STE3-2 (partial)*	*KU315751*
Phaffia rhodozyma CBS 6938	*STE3-2*	*CED85379.1*
Phaffia rhodozyma CBS 7918	*STE3-2*	*PRJNA306035/ LSVH01000253.1 (7004..8395)*
Phaffia rhodozyma CRUB 1149	*STE3-2*	*PRJNA307837/ NODE_198(2416..3807)*
Phaffia rhodozyma KBP 2604	*HD1 (partial)*	*KU315766*
Phaffia rhodozyma ATCC 24261	*HD1 (partial)*	*KU315764*
Phaffia rhodozyma NRRL Y-17434	*HD1 (partial)*	*KU315767*
Phaffia rhodozyma ZP 922	*HD1*	*KU315769*
Phaffia rhodozyma ZP 869	*HD1*	*KU315768*
Phaffia rhodozyma GY13L04	*HD1*	*KU315765*
Phaffia rhodozyma CRUB 1151	*HD1*	*KU315770*
Phaffia rhodozyma ATCC 24229	*HD1*	*KU315763*
Phaffia rhodozyma ATCC 24201	*HD1*	*KU315762*
Phaffia rhodozyma CBS 6938	*HD1*	*CDZ96688.1*
Phaffia rhodozyma CBS 7918	*HD1*	*PRJNA306035/ LSVH01000060.1 (57836..59696)*
Phaffia rhodozyma CRUB 1149	*HD1*	*PRJNA307837/NODE_63 (58233..58452)*
Phaffia rhodozyma ZP 922	*HD2*	*KU315773*
Phaffia rhodozyma ZP 869	*HD2*	*KU315772*
Phaffia rhodozyma GY13L04	*HD2*	*KU315775*
Phaffia rhodozyma CRUB 1490	*HD2*	*KU315780*
Phaffia rhodozyma CRUB 0853	*HD2*	*KU315779*
Phaffia rhodozyma ATCC 24229	*HD2*	*KU315778*
Phaffia rhodozyma ATCC 24201	*HD2*	*KU315777*
Phaffia rhodozyma KBP 2604	*HD2*	*KU315776*
Phaffia rhodozyma ATCC 24261	*HD2*	*KU315771*
Phaffia rhodozyma NRRL Y-17434	*HD2*	*KU315774*
Phaffia rhodozyma CBS 6938	*HD2*	*CDZ96689.1*
Phaffia rhodozyma CBS 7918	*HD2*	*PRJNA306035/ LSVH01000060.1 (55622..57058)*
Phaffia rhodozyma CRUB 1149	*HD2*	*PRJNA307837/ NODE 63 (55665..56306)*
C. neoformans var neoformans JEC21	*STE3*	*XP_570116.1*
C. neoformans var neoformans JEC20	*STE3*	*AAN75624.1*
C. neoformans var grubii H99	*STE3*	*XP_012049557.1*
C. neoformans var grubii 125.91	*STE3*	*AAN75156.1*
C. gattii WM276	*STE3*	*XP_003196044.1*
C. gattii E566	*STE3*	*AAV28758.1*
C. flavescens CF05-CBS8359	*STE3*	*CDR19326.1*
C. flavescens CF01-CBS4918	*STE3*	*CDR19282.1*
K. magrovensis CBS8507	*STE3*	*CCM73226.1*
K. magrovensis CBS10435	*STE3*	*ASQD01000019.1*
K. heveanensis CBS569	*STE3*	*ACZ81463.1*
K. heveanensis BCC8398	*STE3*	*ASQB01000005*
Dioszegia cryoxerica	*STE3*	*ANT03-071JGI-262391*
Dioszegia cryoxerica	*STE3*	*ANT03-071JGI-351885*
Tremella fuciformis tr26	*STE3*	*LBGW01000351*
T. mesentarica ATCC24925	*STE3*	*ADO17672.1*
Sporidiobolus salmonicolor CBS490	*STE3*	*ADM24775.1*
Sporidiobolus salmonicolor CBS483	*STE3*	*ADM24772.1*
Leucosporidium scottii CBS5931	*STE3*	*CRX79175.1*
Leucosporidium scottii CBS5930	*STE3*	*CRX79175.1*
Saccharomyces cerevisiae S288c	*STE3*	*NP_012743.1*
Phaffia rhodozyma CBS 7918	*MFA1*	*PRJNA306035/ LSVH01000226 (14268..14399)*
Phaffia rhodozyma CBS 6938	*MFA1*	*PRJEB6925/ LN483332.1 (1417935..1418060)*
Phaffia rhodozyma CBS 7918	*MFA2*	*PRJNA306035/ LSVH01000253 (9521..9646)*
Phaffia rhodozyma CBS 6938	*MFA2*	*PRJEB6925/ LN483332.1 (1423341..1423472)*
