# Supplementary material for: Genetic Dissection of Sexual Reproduction in a Primary Homothallic Basidiomycete
Source: PLoS Genet. 2016 Jun 21;12(6):e1006110. doi: 10.1371/journal.pgen.1006110 (PMC4915694; doi:10.1371/journal.pgen.1006110)
Supplement: S1 File — (PDF) [file pgen.1006110.s020.pdf]

S1 File. Sequence of Synthetic HD1 and HD2 genes

HD1 (cloned into *Eurofins in-house standard vector pEX-K4-HD1)*

ATGGAACCGAGTCCGGCGAGTGACCTGTCAGCGCGTCTTGACAGTCTCGTGGAAACGTTCCTGATCAGCATTCGC
GCGGGAGTGCCGATCGAATTATCCCAGATCTACGATGCGTTCGCCGAAGAACGCTGCAAAGCGCGTTCTCAAGGA
CTGCTGACCTTCGCCTTTCAGGAAGAAATCTCCGCTTCAGTTGGCTTTCTCTCGACTCTCGCCTCGTGCATGATGAA
TATTCCGGAAGGCAACAAAGACTTTCAGACCACCGTCATTGGCATCTTAAACAATGCAAACAATACGCCAGAGCTGT
GTCCTAGCCGCAACTTAGTTTCCCCTCCGAAGATTCTGGCATCGAAAGCGGATTCGGGCCGCTCGATGCGTCCAC
GCACAGAGCATC**CCTCTTCGTTTCGGCATCTGCGCGATTGGTTCATTGTGCATCTGGATCATCCGTATCCTTCTGC**
TCAGGAGAAAGAGGAGCTTGCTCTGGCACGTAACCTGACCAAGAACTCGATCAACCTGTGGTTCAATAATATGC
GTCGTCGCAGCGGCTGGATGGATTTCGTACGCACACACGCGTTGAACAATCAGACCCGCATGAAAGAACTGGTGT
TGCTTCACCGCTCTCTGCCTGCCGTTGAACGTTCACATCCGGACCTTCCGTTTGAGCTGCGGCAGCAGCTCGATC
GCATTCGCGATCATGTGGATGAGAAAGGTCATGATCGCGTGCGTGATTGGATGACAGGTGCACTGGACTACAAAG
ACGGTTTTGGGGTCCCCGCCGAAGGAATTGGCCGTAGCCCCGAAAAGAAGAACGAAGTCAACAAAGATACCGCGA
AAAGCGGTTCCAGCTCCATCTTGTACAAACAAGCCTTTACTGCGAAAGATGCACAGCCGTGGCTGACCAGTTACCC
CACCGCCGCTAGCGGCTTGATTGGCTATCCGAGTTCAATCCCGCTGGCGAATTGTCCGACGCCGAAAAGTAGCAA
ACGGCTGTTTTCGGATTGTGGTCCGCACTTAGCTGCACCGATTGACCCGGTCAACAAATACCACCAGTTAATTAAC
CTCAGCTCAGGTCCTACCCAGGATCTGGAGGGGTCTTTGCTGACTCACCAACCATTGAGCGCGTATGCTTACTATC
CTACTCATCCGCAATCCCAGAATCCGTTACCAATGCTGGGTCCCACACCGTATCGTTCAAGCAGCGATTCGCAGTA
TCCAGCCTATGACCTGAAAGCACCGATTCGCGCGCAAACCTGGCTGCCGTCTGACCCTGCGATTGATCCGTCAAAT
GGGCCAAACATTCACCAACGTAGCGTTTCCCCATTACCAAGCCGTGCGAAAAGCACGGTATCCTCTCTGGCAACGA
GTACCACGCTCCAAAGCACGCGCTTTTCTGTGAATGAAAGTGCCGGTAATGAACTGGCGGCCAAACTGGAAGCCC
TTCGCATCAAGAAAGAAGCGCTGAAGAAAGAGAAAGAACAGTTGCGCGCCGAAGCTGAAGCACTGGGCGAAGTAT
TCGACTGCTAA

HD2 (cloned into *Eurofins in-house standard vector pEX-K4-HD2)*

ATGTATAGCTTCACCAAGTTGCCGCAGAAATACACTGGTTTAGGAGATGTCTTTGATTCTAACATTGACAGTTTGAG
CAGCAAAGAGTTTGCTCTCGAAGATCTGTATCTGCCAATCCCGACGTCCATTTGCGAGACTTTGGAGGAACTGGGT
ACGCCGCATACCCTGGCCCGCAATTTAGGCGCCCTGTACCTGAGTCGTGCGATGGCGCTGCGCGAACTGAGTTTA
TCGCAGTTTGACACCGCATTCCCTGCCGAACGCACGTTTCTCTTGGAAGCCAACTCCCTCAAACTGAAAGTGTTTAA
AGGCCTGCTTAAAGCGACTCTGGAAAATCGCTACAGCATGGAACTGTTTCACCATCACGAAACCATCATTCAGCTG
GCCGGTGTCTGGACCT**CAGACGATGAACAAGCACCAGTATCCGTACGCCGTAGCTCATTCACGAAGAATACGAT**
TGACACCCTCCAAGCCCTGTATGACCTGAACCAATATCCGAACCCGATTGAGCTGAAAACCATTGCAGTTAAAGT
GGGCTTACAGCCGAAACAGATTCGTGCTTGGTTTCAGAATCGCCGTAATCGGCGCTCTAGCCCCGCACGGTCCC
ATAAGAAACCCCTTAAAATCTCTACCCGTGCTACTGTTGCGAGTGACCTGAAGTTCAACAGCGTCCGCAAACGTTGT
TCGCGCCGTACTACAAGCATCGAAAAGGAGCGCAAAACCGAGGGTGTGCCGTCACAACTGGGCTCACCGATCTTC
TTGCAGACCAACAAAACCCCATACGTTCCTCAGTCGGAAGGCCGCTCTCGCGTTGGTAATTCGTTAGTGGCAGGAC
TGTTCGATACATCGCCCAACGAACAGGATGCGTATGCGCTTTGGAGTGCCGTTGTGAGCGATCGTAGTAGCGCATA
TGCGAACAGTAAGCATGTGCCTCAGATCGAACCATCTCTGGCGTTTGCCATTCCGGAACCGGCGGATAGCATGTC
CGCGCAACGTTCGGAGAATCACTTCAATTCCTGCTCGAAATCGTCCAGTAGCCATCCGGCGTTTCCTCCGGTGTTT
GGGTCTAGCGAAACGATGGAAGTCGATCCGGTAGATTGGACCATGGTTTTCGCGGCTTCAGACTCTCGCAATATTG
CACCACGTAGCGAGAAACCGTCAGCTCGCTGGGAAACGAAAACAGGCCTTGAAGTCGAAGATGCCATGCTGGATG
TGAACCTGAACATGACAACGGGGAAATAA

Note:
Underlined sequence: N-terminal region
Bold sequence: Homeodomain region
